# An MltA-Like Lytic Transglycosylase Secreted by Bdellovibrio bacteriovorus Cleaves the Prey Septum during Predatory Invasion

**DOI:** 10.1128/jb.00475-22

**Published:** 2023-04-03

**Authors:** Emma J. Banks, Carey Lambert, Samuel S. Mason, Jess Tyson, Paul M. Radford, Cameron McLaughlin, Andrew L. Lovering, R. Elizabeth Sockett

**Affiliations:** a School of Life Sciences, Faculty of Medicine and Health Sciences, University of Nottingham, Queen’s Medical Centre, Nottingham, United Kingdom; b School of Biosciences, University of Birmingham, Birmingham, United Kingdom; Geisel School of Medicine at Dartmouth

**Keywords:** *Bdellovibrio bacteriovorus*, predatory bacteria, lytic transglycosylase, MltA, peptidoglycan, bacterial cell division, cell wall, bacterial septum, peptidoglycan hydrolases

## Abstract

Lytic transglycosylases cut peptidoglycan backbones, facilitating a variety of functions within bacteria, including cell division, pathogenesis, and insertion of macromolecular machinery into the cell envelope. Here, we identify a novel role of a secreted lytic transglycosylase associated with the predatory lifestyle of Bdellovibrio bacteriovorus strain HD100. During wild-type B. bacteriovorus prey invasion, the predator rounds up rod-shaped prey into spherical prey bdelloplasts, forming a spacious niche within which the predator grows. Deleting the MltA-like lytic transglycosylase Bd3285 still permitted predation but resulted in three different, invaded prey cell shapes: spheres, rods, and “dumbbells.” Amino acid D321 within the catalytic C-terminal 3D domain of Bd3285 was essential for wild-type complementation. Microscopic analyses revealed that dumbbell-shaped bdelloplasts are derived from Escherichia coli prey undergoing cell division at the moment of Δ*bd3285* predator invasion. Prelabeling of E. coli prey peptidoglycan prior to predation with the fluorescent D-amino acid HADA showed that the dumbbell bdelloplasts invaded by B. bacteriovorus Δ*bd3285* contained a septum. Fluorescently tagged Bd3285, expressed in E. coli, localized to the septum of dividing cells. Our data indicate that B. bacteriovorus secretes the lytic transglycosylase Bd3285 into the E. coli periplasm during prey invasion to cleave the septum of dividing prey, facilitating prey cell occupation.

**IMPORTANCE** Antimicrobial resistance is a serious and rapidly growing threat to global health. Bdellovibrio bacteriovorus can prey upon an extensive range of Gram-negative bacterial pathogens and thus has promising potential as a novel antibacterial therapeutic and is a source of antibacterial enzymes. Here, we elucidate the role of a unique secreted lytic transglycosylase from B. bacteriovorus which acts on the septal peptidoglycan of its prey. This improves our understanding of mechanisms that underpin bacterial predation.

## INTRODUCTION

Dynamic modification of the bacterial peptidoglycan (PG) cell wall is essential for cell growth, division, and bacterial replication (1). Bacteria encode a large, diverse suite of cell wall-modifying enzymes to facilitate these processes, including lytic transglycosylases (LTs). LTs catalyze the nonhydrolytic cleavage of β-1,4-glycosidic bonds between alternating Glc*N*Ac and Mur*N*Ac sugar moieties within the PG wall backbone (2).

LTs fulfil a wide range of roles in bacteria, including PG recycling (3), sporulation (4), division (5, 6), pathogenesis (7–9), and mitigation of periplasmic crowding (10). LTs may also facilitate the insertion of new PG precursors into the cell wall (2) and insertion of appendages such as flagella and pili into the cell envelope (11–13). Escherichia coli K-12 MG1655 encodes nine identifiable different LT proteins: seven within MltA-G, plus Slt70 and RlpA (14). Due to strong functional redundancy, the specific role of most individual LT proteins has been difficult to determine experimentally; however, Slt70 has been linked to PG integrity and repair (15), and MltG generates short glycan strands during PG synthesis (16).

Bdellovibrio bacteriovorus is a small, vibrioid-shaped predatory bacterium that preys upon a wide range of Gram-negative bacteria, living within the prey periplasm (17). No single gene resistance has been identified within prey bacteria throughout many decades of study, highlighting the therapeutic potential of B. bacteriovorus as a novel antibacterial agent (18). B. bacteriovorus attaches to and physically invades its prey, residing within the inner periplasmic compartment of the prey envelope between the prey wall and cytoplasmic membrane (19, 20). The predator then consumes the contents of the dead prey cell, utilizing the nutrients to elongate as a curving filament (21–23). Following nutrient exhaustion, B. bacteriovorus divides to yield multiple daughter cells which lyse the prey cell and seek new prey to invade (24).

B. bacteriovorus uses many PG enzymatic tools to facilitate this dual bacterial encounter (25–28), including two DD-endopeptidases which are involved in prey invasion. These two enzymes, Bd0816 and Bd3459, are secreted by B. bacteriovorus into the prey periplasm and cut cross-links between PG muropeptides in the prey cell wall (29). Relaxation of prey PG results in the transformation of rod-shaped prey into spherical prey “bdelloplasts” (29). The action of LD-transpeptidase enzymes, including Bd0886 and Bd1176, adds additional cross-links to these modified prey PG muropeptides forming a stable niche in which B. bacteriovorus sequentially degrades the prey cell contents and replicates (26).

The genome of the B. bacteriovorus-type strain, HD100, encodes numerous additional PG-active enzymes, many of which have not yet been studied. These include at least thirteen putative LT genes of unknown function (30). Three such genes, *bd0519, bd0599*, and *bd3285*, share homology with the LT protein MltA in E. coli K-12 MG1655 (hereafter referred to as MltA_Ec_). Unlike most LTs, MltA proteins have a β-barrel endoglucanase V-like fold (31). MltA_Ec_ is a kidney-bean shaped monomeric protein that contains a lipobox signal and the domains “A” and “B” (31). Domain A contains the catalytic 3D domain, named for three important aspartate residues, D281, D317, and D328 (31). While purified MltA_Ec_ has been shown to cleave PG *in vitro* (31), the *in vivo* role of MltA_Ec_ is unclear as an E. coli Δ*mltA* mutant did not reveal a phenotype under laboratory conditions (32). Deletion of the *Neisseria* sp. MltA homologue, LtgC, however, resulted in daughter cells that were defective in cell separation (33, 34).

Cleavage of prey PG backbones is important during predation to allow predator entry into and exit from the prey cell (27). We therefore examined the roles of the three B. bacteriovorus-encoded MltA-like proteins Bd3285, Bd0519, and Bd0599 during predation. We discovered that deletion of *bd3285* gave a predator that produced a mixture of bdelloplast shapes when preying upon E. coli: spheres, rods, and “dumbbells.” We further showed that dumbbell-shaped bdelloplasts originated from dividing prey cells and that these contained an intact PG septum that was not seen in wild-type predatory cultures which produced spherical bdelloplasts. These findings indicate that the bacterial predator B. bacteriovorus secretes a lytic transglycosylase into the prey periplasm to cleave the PG septum of dividing prey, facilitating the conversion of the whole dividing prey cell into a spherical bdelloplast niche.

## RESULTS

### Predator *mltA*-like lytic transglycosylase genes are upregulated during prey invasion.

**FIG 1:**
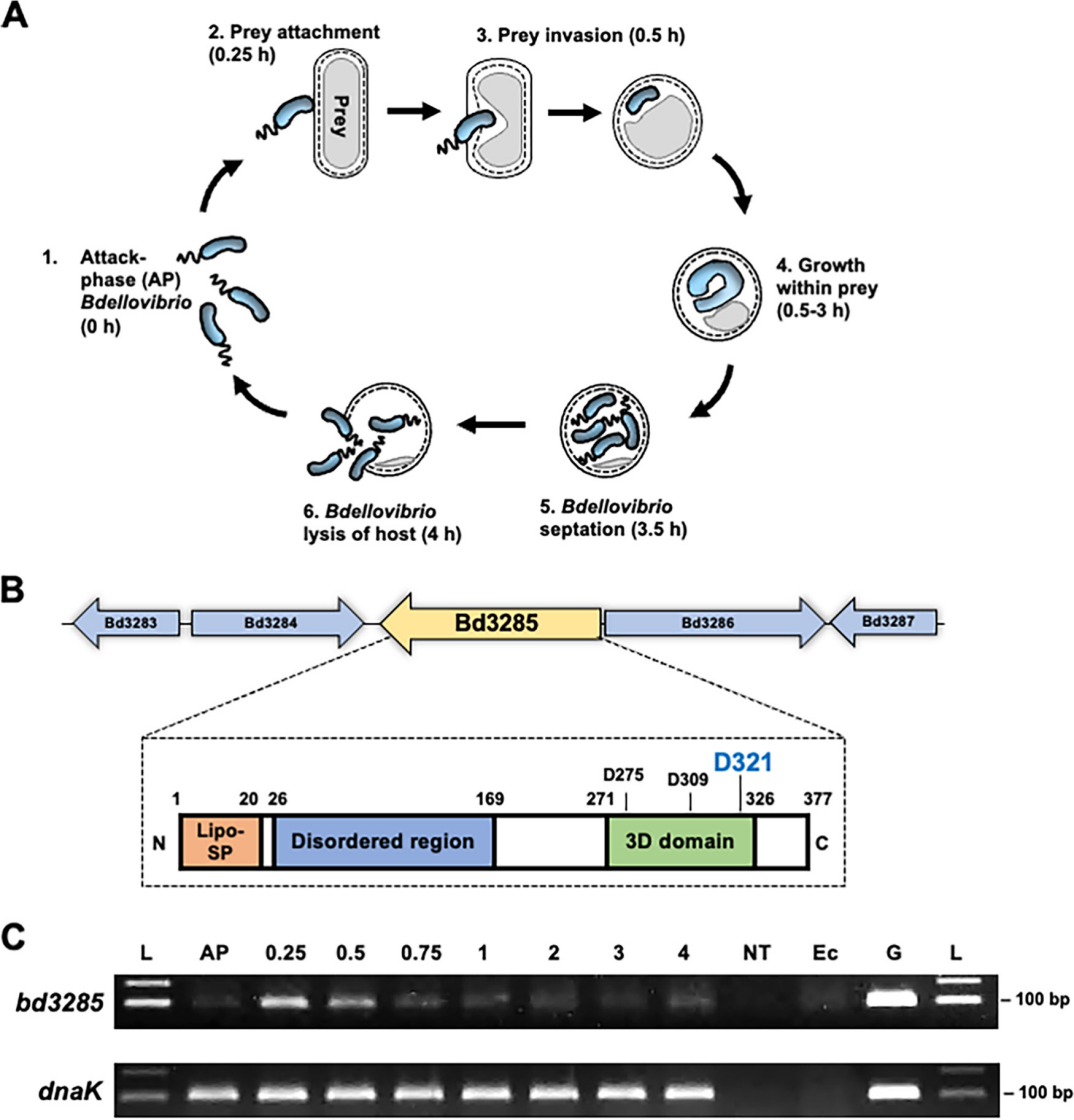
Upregulation of *bd3285* during prey invasion by Bdellovibrio bacteriovorus and schematic of the encoded protein. (A) Predatory life cycle of Bdellovibrio bacteriovorus. (1) Attack-phase (AP) B. bacteriovorus cells swim or glide to locate Gram-negative prey bacteria to which they attach (2) and then physically invade, concurrently rounding the rod-shaped prey cell into a spherical prey “bdelloplast” (3). B. bacteriovorus elongates as a filament within the prey periplasm, consuming the nutrients of the dead prey (4) until nutrients are exhausted and the predator divides to yield multiple progeny cells (5). B. bacteriovorus progeny then lyse the dead prey cell and seek out new prey to invade (6). (B) Genetic locus of the monocistronic *bd3285* gene within the genome of B. bacteriovorus HD100 (top) and a schematic of the predicted domains and regions of the Bd3285 protein (bottom). Lipo-SP, lipoprotein signal peptide; 3D domain, domain containing 3 aspartate residues (D275, D309, and D321) predicted to be critical for catalysis based on homology to E. coli K-12 MG1655 MltA. (C) Reverse transcriptase PCR performed on B. bacteriovorus HD100 RNA isolated at time points during a predatory cycle on E. coli S17-1 prey. *dnaK* is a constitutively transcribed control gene. L, molecular weight 100 bp ladder; AP, attack-phase predators; 0.25 to 4, hours since predation commenced; NT, no template RNase-free water; Ec, E. coli S17-1 RNA, G, B. bacteriovorus HD100 genomic DNA. Data are representative of three biological repeats.

The predatory life cycle of B. bacteriovorus comprises distinct stages (Fig. 1A) involving specialized predator effector proteins—many of which remain to be characterized. The genome of B. bacteriovorus type strain HD100 encodes three homologues of the E. coli K-12 MG1655 LT protein MltA (MltA_Ec_): Bd3285, Bd0519, and Bd0599. Gene *bd3285* encodes a 377 amino acid (aa) protein, similar in length to MltA_Ec_ (365 aa), while Bd0519 and Bd0599 proteins are shorter (247 aa and 237 aa, respectively) (Fig. S1). Like MltA_Ec_, Bd3285 contains a predicted lipoprotein signal peptide (aa 1 to 20) and a C-terminal 3D (three aspartate) domain (aa 271 to 326) (Fig. 1B). The three important aspartate residues of the MltA_Ec_ catalytic 3D domain are conserved in Bd3285, including residue D321 (MltA_Ec_^D328^) which is crucial for the catalytic function of MltA_Ec_ (31) (Fig. 1B; Fig. S2). Unique to Bd3285, and following the lipoprotein signal peptide, is a long, disordered N-terminus (aa 26 to 169) (Fig. S3). This region is absent from MltA_Ec_ and also from Bd0519 and Bd0599 which solely encode a 3D domain, each containing the three conserved aspartate residues (Fig. S1; Fig. S2).

Upregulation of B. bacteriovorus gene expression at a particular time point during predation is often indicative of a particular predatory function; the DD-endopeptidase genes *bd0816* and *bd3459* are both upregulated at 0.25 to 0.5 h, consistent with their role in sculpting rod-shaped prey into spherical bdelloplasts during prey invasion (29) (Fig. 1A). Reverse-transcriptase PCR with *mltA_Bd_*-specific primers on RNA isolated from different time points during a predatory cycle on E. coli S17-1 prey showed that all three B. bacteriovorus *mltA*-like genes were upregulated 0.25 to 0.5 h into the predatory life cycle (Fig. 1C for *bd3285*; Fig. S4 for *bd0519*; Fig. S5 for *bd0599*), perhaps suggesting a role for these proteins in prey invasion processes.

### Bd3285 is important for the transformation of prey shape during invasion.

To determine whether the B. bacteriovorus MltA-like LTs are involved in prey invasion, markerless deletions of each gene were constructed and verified within the genome of B. bacteriovorus HD100. To visualize B. bacteriovorus predators inside prey bdelloplasts during predation, a fluorescent fusion of Bd0064-mCherry (a cytoplasmic PilZ domain-containing protein, present throughout predation) was introduced into the genomes of B. bacteriovorus wild-type and deletion mutant strains by single-crossover homologous recombination. Fusions of fluorophores to the C-terminus of Bd0064 have been used extensively in previous studies to successfully label the cytoplasm of predator cells without adverse effects (28, 35, 36).

**FIG 2:**
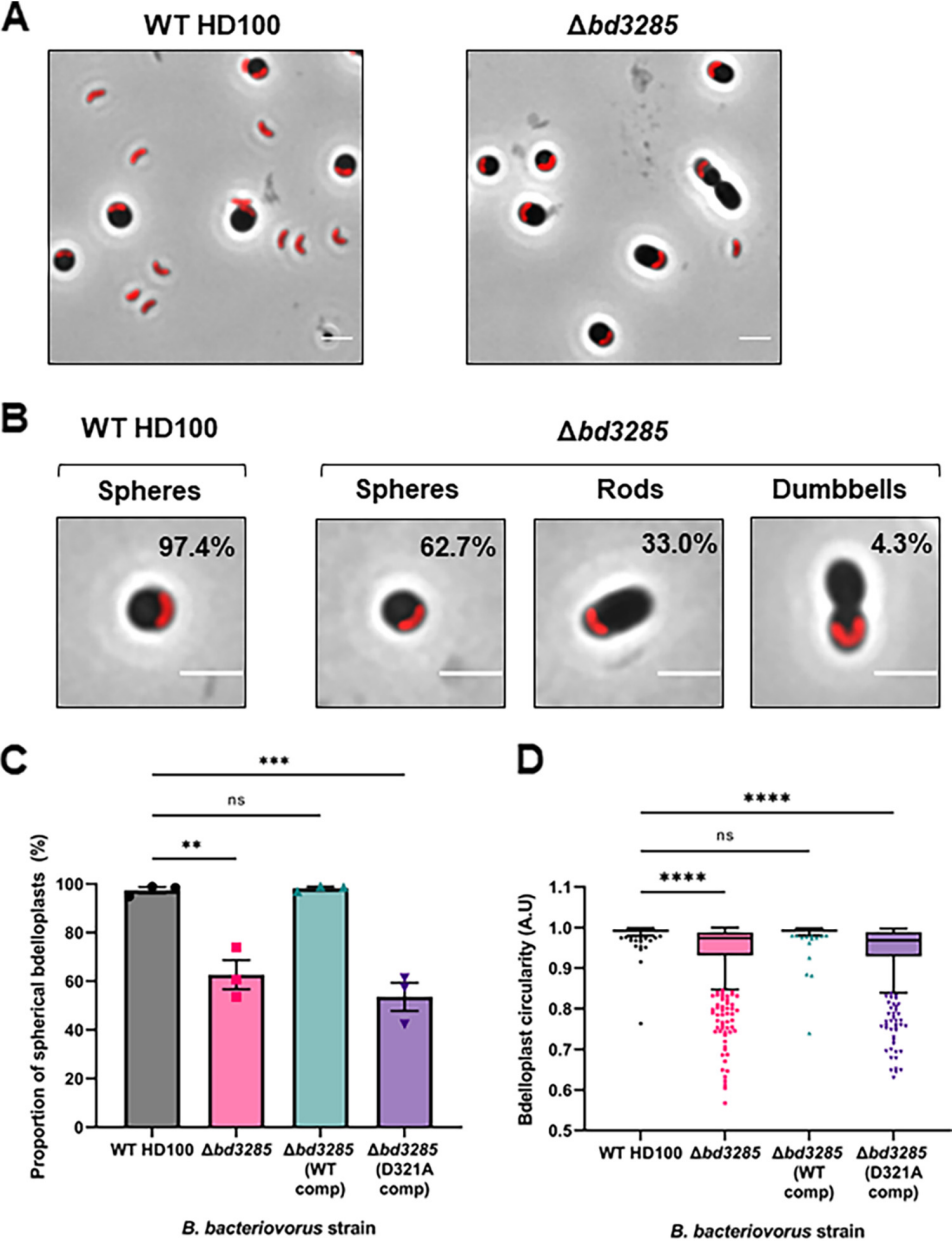
Deletion of *bd3285* gives prey bdelloplasts with differing morphologies from E. coli prey. (A) Predation of B. bacteriovorus HD100 wild-type or Δ*bd3285* upon E. coli S17-1 prey showing the different morphologies of prey bdelloplasts invaded by each strain. (B) Magnified images highlighting the three different bdelloplast shapes for prey invaded by Δ*bd3285* compared to uniquely spherical prey generated by WT HD100 invasion. The proportion of bdelloplasts represented by each shape is denoted in the top right of each image. B. bacteriovorus predator cells are labeled in red via the fluorescent fusion of cytoplasmic protein Bd0064-mCherry. Scale bars = 2 μm and images are representative of three biological repeats. (C) Proportion of spherical bdelloplasts (classified as spherical by a circularity value of >0.96 A.U.) invaded by wild-type, Δ*bd3285*, Δ*bd3285* (WT comp), or Δ*bd3285* (D321A comp) predators. Error bars represent SE of the mean. Ns, nonsignificant; **, *P* < 0.0018; ***, *P* < 0.0004; (one-way ANOVA with Tukey’s multiple-comparison test). (D) Circularity of bdelloplasts invaded by the same strains. Box, 25th to 75th percentiles; line, median; whiskers, Tukey; ns, nonsignificant; ****, *P* < 0.0001; (Kruskal-Wallis test with Dunn’s multiple-comparison test). For C and D, *n* = 234 to 670 cells were analyzed at the 1 h predation time point from three biological repeats.

Wild-type and deletion mutant predator strains were mixed with stationary-phase E. coli S17-1 prey to initiate a predatory time course and images were acquired at different time points. Notably, in contrast to E. coli prey invaded by wild-type predators which all rounded up into spherical bdelloplasts, E. coli prey invaded by the Δ*bd3285* mutant formed three distinct shapes: spheres, rods, and rods with a midcell constriction (hereafter referred to as “dumbbells”) (Fig. 2A). While rod-shaped bdelloplasts have been observed before during predation on E. coli prey with a DD-endopeptidase Δ*bd0816/*Δ*bd3459* predator mutant (29), the dumbbell shapes observed in predation with the Δ*bd3285* mutant were unique.

Quantification of bdelloplast shapes revealed that 97.4% ± SD 2.2% of E. coli bdelloplasts invaded by wild-type predators were spherical, while 2.6% ± SD 2.2% were rod-shaped. In contrast, for E. coli invaded by Δ*bd3285*, 62.7% ± SD 10.4% of bdelloplasts formed spheres, 33.0% ± SD 8.6% formed rods, and 4.3% ± SD 1.8% formed dumbbells (Fig. 2B). The proportion of spherical bdelloplasts was significantly lower (*P* < 0.0018) for Δ*bd3285* predation compared to the wild-type (Fig. 2C), illustrated by bdelloplast median circularity measurements (*P* < 0.0001; Fig. 2D). E. coli prey bdelloplasts produced by Δ*bd3285* invasion were also both significantly longer and narrower than those invaded by the wild-type strain (*P* < 0.0001; Fig. S6), reflecting the presence of rod- and dumbbell-shaped bdelloplasts caused by Δ*bd3285* mutant predators which are not seen during wild-type predation.

Complementation of the Δ*bd3285* predator mutant by double-crossover homologous reintegration of the wild-type *bd3285* gene (WT comp) completely restored wild-type spherical bdelloplast morphology (Fig. 2C and D). In contrast, complementation of Δ*bd3285* with a copy of *bd3285* containing a mutation of the predicted catalytic aspartate residue D321 (D321A comp), failed to restore wild-type spherical bdelloplast shape, indicating that the 3D domain residue D321 is critical for Bd3285 function (Fig. 2C and D). Stability of the Bd3285 (D321A comp) allele was verified by Western blotting analysis of an mCherry-tagged version with the wild-type Bd3285-mCherry (Fig. S7).

However, neither of the other MltA_Bd_ proteins, Bd0599 or Bd0519, appeared to affect bdelloplast shape transformation, because during predation with either Δ*bd0599* or Δ*bd0519* mutant predators, prey cells rounded up into spherical bdelloplasts (100% ± SD 0% for Δ*bd0599* and 97.4% ± SD 1.3% for Δ*bd0519* (Fig. S8)).

Deletion of *bd0519* resulted in delayed prey cell entry with only 68.9% ± SD 8.0% of prey cells containing a B. bacteriovorus predator 30 min after predator-prey mixing compared to 92.7% ± SD 3.0% for the wild-type (Fig. S9). There was no significant defect in prey entry for Δ*bd0599* or Δ*bd3285* mutant predators, with 94.6% ± SD 2.0% and 95.5% ± SD 2.5% of prey cells invaded at 30 min, respectively (Fig. S9). The delay in prey entry for the Δ*bd0519* mutant resulted in a slightly prolonged predatory cycle with more bdelloplasts still visible 5 h after predator-prey mixing in comparison to the wild-type (Fig. S9). Thus, both other MltA_Bd_ proteins have evolved different functions in the predatory life cycle, with the function of Bd0599 remaining unclear and Bd0519 likely to be involved in prey cell invasion, possibly contributing to (but not solely responsible for) porthole formation.

### Dumbbell-shaped bdelloplasts are derived from dividing E. coli prey.

As the shape of dumbbell bdelloplasts from Δ*bd3285* predation resembled that of dividing E. coli, we hypothesized that dumbbell bdelloplasts originate from E. coli cells that were undergoing cell division at the moment of invasion by B. bacteriovorus. Invasion by Δ*bd3285* (concurrent with the death of the dividing prey cell) would result in persistence of the septating E. coli shape, forming a dumbbell bdelloplast.

**FIG 3:**
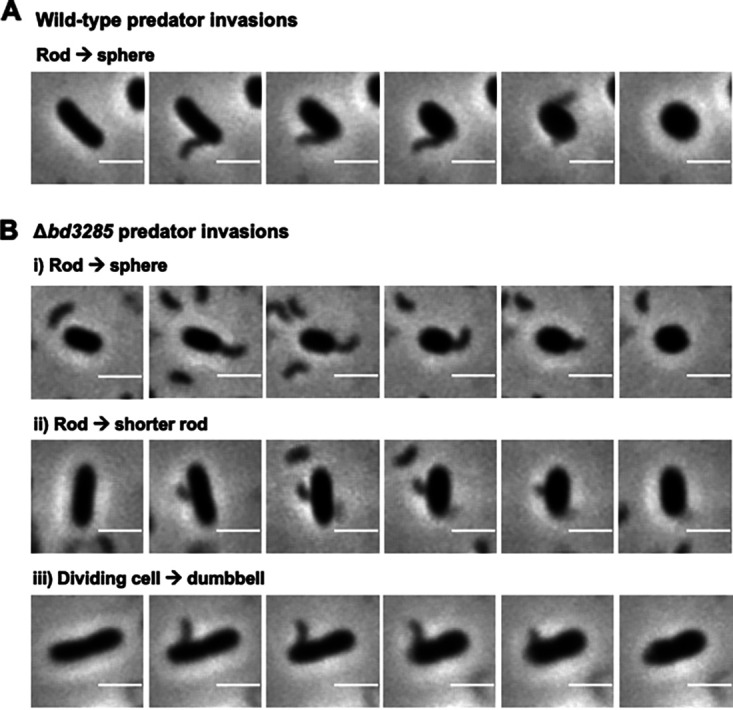
Prey shape transformation by wild-type and Δ*bd3285* B. bacteriovorus strains. Time-lapse microscopy stills of prey invasion by either B. bacteriovorus HD100 wild-type (A) or Δ*bd3285* (B) into E. coli S17-1 prey. Three examples of prey invasion for Δ*bd3285* are shown: (i) a rod rounding up into a spherical bdelloplast; (ii) a rod shortening in length but remaining rod-shaped; and (iii) a dividing E. coli that shortens in length and becomes a dumbbell bdelloplast. Scale bars = 2 μm and examples are representative of three biological repeats.

In support of this hypothesis, there was no significant difference (*P* = 0.10) between the proportion of E. coli cells dividing prior to predation (6.8% ± SD 1.1%) and the proportion of dumbbell bdelloplasts formed during predation by B. bacteriovorus Δ*bd3285* (4.3% ± SD 1.8%) (Fig. S10). Time-lapse microscopy visualized individual predatory invasion events and, as predicted, wild-type predators sculpted rod-shaped E. coli cells into spheres during invasion (Fig. 3A). During invasion by Δ*bd3285* predators, rod-shaped prey were either sculpted into spheres or shortened in length but remained rod-shaped. Dividing E. coli prey that were invaded by Δ*bd3285* predators shortened slightly with some rounding at the cell poles, and the fixed septum producing a dumbbell shape (Fig. 3B).

These data suggest that the unique dumbbell-shaped bdelloplasts observed in the Δ*bd3285* mutant are formed from E. coli prey cells that were undergoing cell division until invasion by B. bacteriovorus, resulting in death of the prey and fixation of dividing cell shape.

### Bd3285 localizes to and cleaves the septum of dividing E. coli prey.

Considering that dividing prey could not be sculpted into spherical bdelloplasts by the Δ*bd3285* mutant, we hypothesized that Bd3285’s wild-type role may be to interact with the peptidoglycan at the septum of dividing prey bacteria. Since Bd3285 contains an N-terminal lipobox-containing signal peptide, it is likely that the protein is secreted from B. bacteriovorus predator cells into the prey periplasm allowing access to the prey septum. Similar secretion patterns from predator into prey have been seen for two other lipobox proteins that modify prey-PG walls: novel lysozyme Bd0314 (DslA) which recognizes deacetylated peptidoglycan (27), and one of the prey-PG Glc*N*Ac deacetylating enzymes Bd3279 (25). We tested this by constructing a fusion of Bd3285-mCherry within the chromosome of B. bacteriovorus in combination with a fusion of Bd0064-mCerulean3 (to label the cytoplasm of predator cells within prey bdelloplasts). During predation of this dually labeled B. bacteriovorus strain on E. coli, we observed that Bd3285-mCherry (while showing very faint signal, which is common for low level, high activity, B. bacteriovorus PG-active enzymes [26]) localized separately from the predator cell within the prey bdelloplast, suggesting that Bd3285 is indeed secreted into prey (Fig. S11). The Bd3285-mCherry fusion also retained functional activity as determined by complementation of the Δ*bd3285* mutant (Fig. S12).

**FIG 4:**
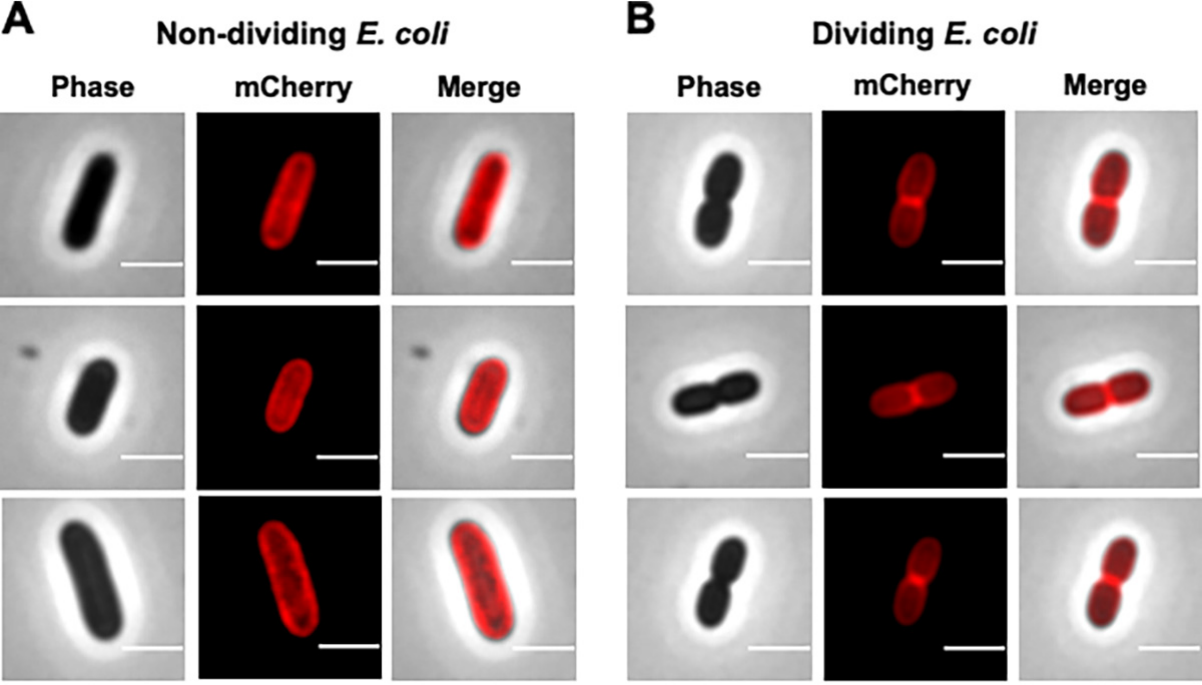
Bd3285-mCherry localizes to the septum of dividing E. coli cells. Fluorescence microscopy images of E. coli TOP10 containing the fusion of Bd3285-mCherry within the vector pBAD. Cells were induced with 0.2% arabinose for 20 h and then images were acquired of both nondividing cells (A) and dividing cells with a visible constriction at the midcell (B). Scale bars = 2 μm and images are representative of three biological repeats.

We also tested the cellular location of Bd3285-mCherry protein heterologously expressed in E. coli TOP10 cells under the control of an arabinose-inducible pBAD vector promoter. Bd3285-mCherry showed heterogeneity in fluorescence brightness, with individual cells varying from no fluorescence to extremely bright fluorescence (Fig. S13). Some cells appeared visibly damaged by overexpression of Bd3285-mCherry which may account for the variation in fluorescence intensity (Fig. S13) but heterologous expression did give an opportunity to examine cellular destinations of the protein. In E. coli TOP10 cells exhibiting optimal brightness, Bd3285-mCherry protein appeared to localize most strongly to the periplasmic compartment (Fig. 4A and B). Most interestingly, in cells that had a constriction at the midcell and were undergoing division, Bd3285-mCherry primarily localized to the midcell septum (Fig. 4B; Fig. S14). It is important to note that the shape of these dividing E. coli TOP10 cells expressing just Bd3285 alone differs from the dumbbell bdelloplasts observed during predation by the Δ*bd3285* B. bacteriovorus mutant (the cell compartments are less rounded). This is because in E. coli TOP10 (Fig. 4), only the Bd3285 protein is being expressed, whereas during predation experiments, a full consignment of predator enzymes are secreted into E. coli prey, including two DD-endopeptidase enzymes which contribute to the sculpting of a rounded prey bdelloplast shape (discussed later in further detail).

**FIG 5:**
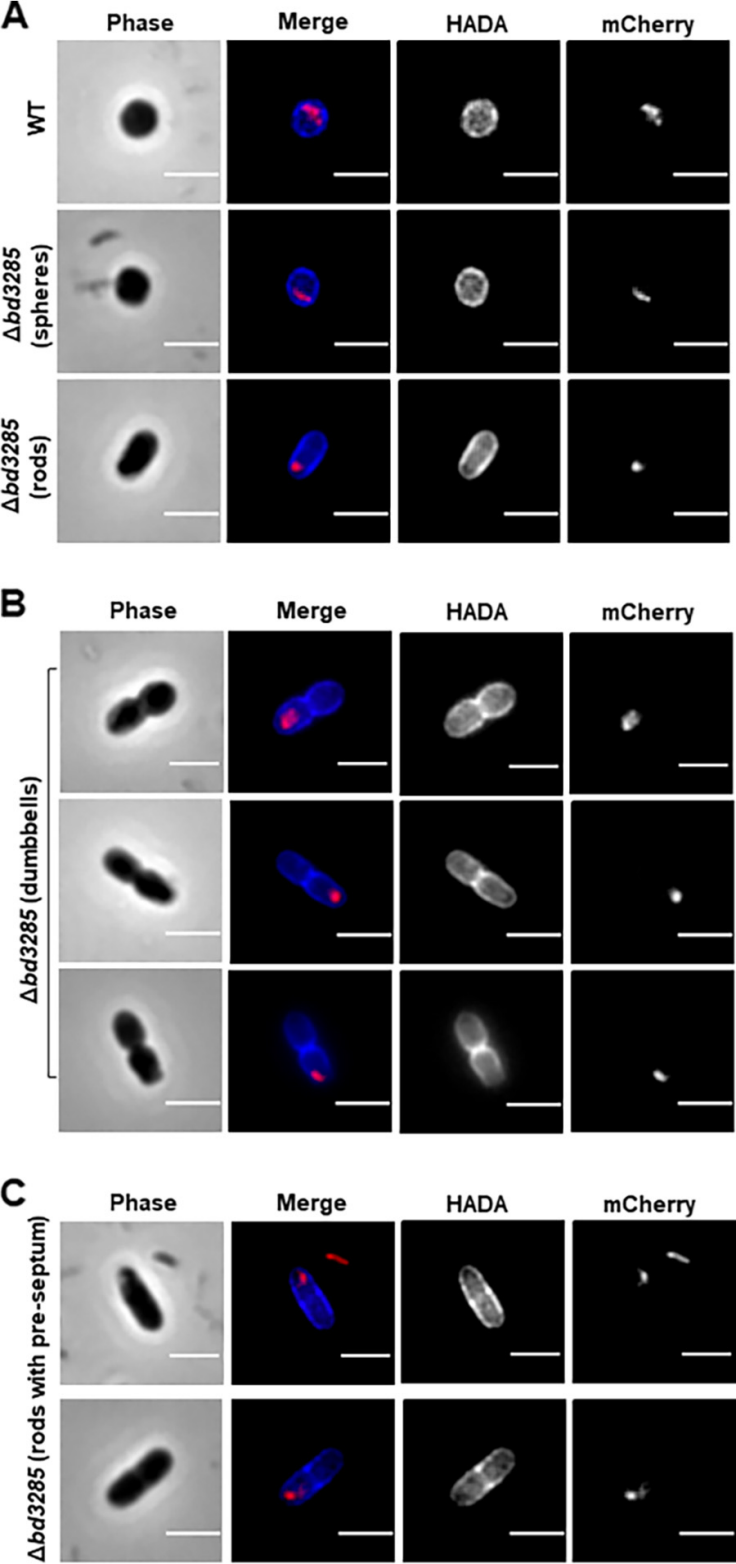
The septum of dividing E. coli is not cleaved in prey invaded by B. bacteriovorus Δ*bd3285.* Fluorescence microscopy images of E. coli S17-1 prey invaded by either B. bacteriovorus wild-type or Δ*bd3285*. The E. coli PG cell wall was prelabeled with the blue D-amino acid HADA prior to predation. B. bacteriovorus predator strains contain a Bd0064-mCherry fusion to label the predator cytoplasm and allow visualization of predators inside prey. Samples were fixed for imaging 30 min after predator-prey mixing. (A) Spherical bdelloplast invaded by a wild-type predator (top row), and spherical and rod-shaped bdelloplasts invaded by Δ*bd3285* (middle and bottom rows, respectively). (B) Examples of dumbbell-shaped bdelloplasts invaded by Δ*bd3285* which still contain a septum. (C) Examples of rod-shaped bdelloplasts that appear to contain some preseptal PG. Scale bars = 2 μm and images are representative of three biological repeats.

As Bd3285 localizes to the E. coli septum and the protein is a predicted lytic transglycosylase, we hypothesized that Bd3285 may cleave the septum of dividing prey cells. If true, then the septum should still be intact within dumbbell-shaped bdelloplasts. To test this hypothesis, we used the fluorescent D-amino acid (FDAA) HADA to prelabel the E. coli PG cell wall prior to predation. B. bacteriovorus wild-type and Δ*bd3285* strains (each containing a Bd0064-mCherry fusion to label the cytoplasm) were incubated with prelabeled E. coli S17-1 for 30 min and then samples were removed for imaging. E. coli bdelloplasts formed by invading wild-type B. bacteriovorus were spherical in shape and blue HADA signal was observed throughout the bdelloplast sphere, with HADA uniformly labeling all PG (Fig. 5A). Spherical and rod-shaped bdelloplasts formed by Δ*bd3285* predators also showed uniform HADA incorporation (Fig. 5A). Critically, all dumbbell-shaped bdelloplasts showed additional fluorescent HADA signal across the midcell, labeling septal PG (Fig. 5B; Fig. S15). Interestingly, some rod-shaped bdelloplasts had HADA signal at the midcell sidewalls which may represent preseptal PG as the prey cell prepares to undergo cell division (Fig. 5C). This preseptal PG might prevent full conversion of these rods into spheres by other predatory enzymes in the absence of Bd3285 (discussed in further detail later).

The complementation strain Δ*bd3285* (WT comp) formed solely spherical bdelloplasts with a uniform HADA signal (Fig. S16A), while predation with the Δ*bd3285* (D321A comp) strain resulted in a mixture of spheres, rods, and dumbbell bdelloplasts (with HADA labeling of the dumbbell septum) (Fig. S16A and B).

Collectively, these data show that the PG septum of dividing E. coli prey cells remains, and is not cleaved upon invasion by Δ*bd3285* predators, indicating that B. bacteriovorus lytic transglycosylase Bd3285 is involved in the cleavage of prey septal PG. Failure to cleave the septum results in prey bdelloplasts that are not sculpted into optimal bdelloplast spheres.

## DISCUSSION

During invasion into Gram-negative prey, the bacterial predator B. bacteriovorus secretes an arsenal of PG-modifying enzymes to modify the prey cell wall, creating an entry porthole through which the predator can access the prey periplasmic compartment. Here, we identify a novel lytic transglycosylase lipoprotein, Bd3285, which is secreted by B. bacteriovorus into E. coli prey to cleave the PG septum of prey cells undergoing cell division.

Three B. bacteriovorus proteins, Bd3285, Bd0599, and Bd0519, share homology with the lytic transglycosylase MltA from E. coli K-12 MG1655, including strong conservation of a C-terminal catalytic 3D domain (Fig. S1). Although MltA_Ec_ has been well characterized structurally and biochemically (31, 37), a role for MltA_Ec_ *in vivo* has not been demonstrated (32). We discovered that all three B. bacteriovorus *mltA*-like genes were upregulated at the time point of prey invasion (Fig. 1C; Fig. S4 and 5). While no phenotype could be determined for Bd0599, prey invasion was delayed in a Δ*bd0519* mutant, but not in the Δ*bd3285* mutant (Fig. S9).

Most striking, however, was the observation that a proportion of prey cells invaded during predation by a Δ*bd3285* mutant did not round up into spherical bdelloplasts, in contrast to invasion by wild-type B. bacteriovorus (Fig. 2) and in contrast to Δ*bd0519* Δ*bd0599* mutants (Fig. S8). Additional experiments revealed that Bd3285 is secreted from the indwelling predator into the prey periplasm (Fig. S11), can localize to a prey septum (Fig. 4), and is required to cleave the septum of these dividing prey during bdelloplast formation (Fig. 5).

The phenotypic diversity of these similar MltA-like LT proteins contrasts with that found for self-LT processes in V. cholerae (10). There, the absence of a specific LT was compensated for by expression of another heterologous LT (10). In B. bacteriovorus, neither the lipobox-containing MltA homologue Bd0599 nor the sec-signal-containing Bd0519 compensate for Bd3285 in prey septal-processing.

Bd3285 secreted by B. bacteriovorus appears to preferentially localize to the prey septum and this is a protein-intrinsic property also seen when Bd3285 is heterologously expressed in E. coli. Septal localization has also been observed for the LT proteins RlpA (in both Pseudomonas aeruginosa [5] and Vibrio cholerae [6]) and MltC in Vibrio cholerae (6). Both proteins are involved in daughter cell separation (5, 6). RlpA contains a SPOR domain which specifically binds septal PG by preferentially recognizing denuded glycan chains at the septum (5, 38, 39). However, Bd3285 does not contain a SPOR domain nor any other domains known to bind differentially modified PG, suggesting that its mode of recruitment and binding to PG may differ from other proteins. It would be interesting to test the catalytic properties of Bd3285 *in vitro*; however, we were unable to produce soluble Bd3285, having been guided by an Alphafold model to clone region S204-K420 into pET29b. Trial expression in BL21 cells yielded a clear signal which unfortunately was entirely in the insoluble fraction and was not amenable to standard refolding protocols.

Bd3285 has a lipobox motif and a disordered N-terminal domain which may be involved in a novel secretion and targeting mechanism. This must initially target Bd3285 to the B. bacteriovorus periplasm, then outer membrane, and from there it may be inserted into either the prey inner or outer membrane, or the lipid anchor may be cleaved to release Bd3285 into the prey periplasm. This may then diffuse to interact with the prey PG, preferentially targeting the septum. An alternative is that Bd3285 may be packaged into outer membrane vesicles that are delivered to the prey; such vesicles have recently been observed in bdelloplasts via cryo-electron tomography (40). As B. bacteriovorus has a wide prey range, this novel targeting would have to be of a general nature rather than by specific interactions with other proteins as these would vary considerably between different prey. It is noteworthy that the timing of expression of Bd3285 and its action on the prey cell septum comes early in the predatory cycle before the predator itself is forming septa.

**FIG 6:**
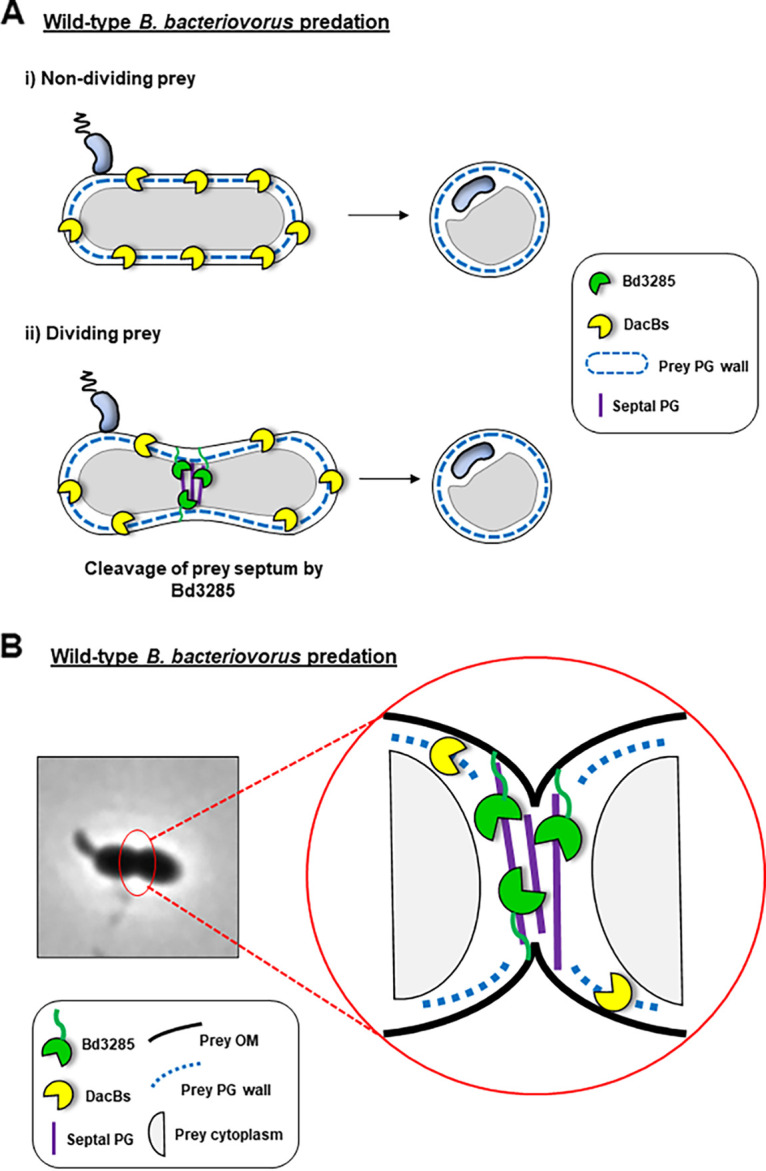
Model for potential Bd3285 activity during wild-type B. bacteriovorus predation. (A) Model of predation of wild-type B. bacteriovorus upon E. coli prey that is either (i) non-dividing or (ii) dividing at the moment of predator invasion. Non-dividing prey does not contain any septal peptidoglycan; therefore, de-cross-linking activity of DacB enzymes alone is sufficient to convert rod-shaped prey into spherical bdelloplasts (i). In dividing prey with septal peptidoglycan, however, Bd3285 lytic transglycosylase action is required to cleave the septum, facilitating full conversion of dividing cells into spherical bdelloplasts (ii). (B) Close-up model of Bd3285 action at the septum during predation on dividing prey cells.

Previous work by Lerner et al. uncovered the role of two DD-endopeptidase (DacB) enzymes which are secreted by B. bacteriovorus during prey invasion (29). Deletion of both *dacB* genes resulted in a population of ~95% rod-shaped bdelloplasts (29). Comparing this to the morphology of bdelloplasts produced by Δ*bd3285* mutant predators where we found that 33.0% formed rods, we suggest that it is likely that Bd3285 has evolved to act in concert with the DacB enzymes to optimize prey invasion by wild-type predators. During predation by Δ*bd3285* predators, DacB activity alone may be sufficient to sculpt most short rod-shaped E. coli cells into spheres as 62.7% of bdelloplasts from the Δ*bd3285* mutant are spherical. For the ~33.0% of rod-shaped prey bdelloplasts from the Δ*bd3285* mutant that shorten in length but remain rod-shaped, it is possible that a ring of preseptal PG (not cut by Bd3285 in Δ*bd3285*) may hold the rod shape in place, preventing complete cell rounding by other enzymes during predation. Equally, for prey cells undergoing a later stage of cell division at the point of predator invasion, septal PG (not cut by Bd3285 in Δ*bd3285*) could also fix this constricted septating cell shape in place, resulting in dumbbell-shaped bdelloplasts. In both of the latter scenarios, it is possible that the DacB enzymes, Bd0816 and Bd3459, can access PG cross-links at the midcell sidewall, but the presence of septal or preseptal PG (that is not cleaved by Bd3285) fixes the prey shape in place, preventing conversion into spherical bdelloplasts. During predation by wild-type B. bacteriovorus, cleavage of septal PG by Bd3285 would therefore be required to facilitate the complete conversion of E. coli rods and dividing cells by other PG-active enzymes (particularly DacBs) into spherical bdelloplasts (Fig. 6). Such a uniformly spherical shape may distribute mechanical stresses, from the predator within, evenly across the bdelloplast envelope, preventing premature lysis prior to predator replication.

Considering the evolution of predation, cleavage of the septum of a prey cell to form a fully rounded bdelloplast likely creates an optimized niche for predator growth, enabling access to the whole prey contents and room for filamentous growth throughout the bdelloplast. Therefore, we suggest that, in addition to generating strong, stable spherical bdelloplasts, another predatory population-fitness advantage conferred by Bd3285 septum-cleavage may be to provide a single invading B. bacteriovorus predator cell access across any prey septa to maximal prey nutrients during intra-bdelloplast growth without a second invasion being required on the distal side of the septum. Unfortunately, we cannot quantify any fitness change experimentally due to the varying and low percentage of septating prey, but we suggest that evolution of Bd3285 may have arisen as an additional “efficiency benefit” to basic predation in this way.

In summary, we have uncovered a novel function for a lytic transglycosylase lipoprotein which is secreted by B. bacteriovorus into prey bacteria to cleave the septal PG of dividing cells, allowing each predator access to the whole prey as a niche, whether a septum is present or not. These findings enhance our fundamental understanding of the life cycle of a bacterial predator that has potential application in future antibacterial therapies.

## MATERIALS AND METHODS

### Bacterial culture.

Bdellovibrio bacteriovorus strain HD100 (Type strain) was routinely cultured in liquid calcium/HEPES buffer (5.94 g/L HEPES free acid, 0.284 g/L calcium chloride dihydrate, pH 7.6) containing Escherichia coli S17-1 (~1 × 10^9^ CFU/mL) in a predator to prey ratio of 1:3 as described previously (41). For growth on solid media, B. bacteriovorus was cultured on double-layer YPSC plates as described previously (41). Kanamycin-resistant E. coli S17-1 (pZMR100) was substituted as prey for the culture of kanamycin-resistant B. bacteriovorus strains. E. coli strains used as bacterial prey were routinely cultured on either solid YT or in liquid YT media, inoculated by a single colony and incubated at 37°C for 16 h, with orbital shaking at 200 rpm. Media were supplemented with kanamycin (25 μg/mL) when required.

### Reverse-transcriptase PCR.

To measure the transcriptional pattern of *mltA*-family genes, versus the *dnaK* constitutively expressed control, reverse transcriptase PCR (RT-PCR) was carried out on RNA template isolated from different time points during predation of B. bacteriovorus HD100 on E. coli S17-1 using an SV Total RNA Isolation System kit (Promega) as described previously (42). The quality of RNA template was verified on an Agilent Bioanalyzer using the Agilent RNA 6000 Nano Kit. RT-PCR was performed using the Qiagen OneStep RT-PCR kit using the parameters: 50°C for 30 min, 94°C for 15 min, followed by 30 cycles of 94°C for 1 min, 50°C for 1 min, and 72°C for 1 min, and a final step of 72°C for 10 min. Samples were visualized on a 2% agarose gel run at 100 V for 20 min.

### Construction of deletion and complementation strains.

Primers and plasmids used to construct strains used in this study are detailed in Table S1 and Table S2. Bacterial strains are listed in Table S3. Bd3285 amino acid numbering was updated to correct for a mis-annotated start codon (the true protein starts at M44; Fig. S14).

To construct in-frame silent genetic deletions in B. bacteriovorus, 0.5 to 1 kb of DNA flanking the gene of interest was cloned into the suicide vector pK18*mobsacB* by Gibson Assembly (43) using the NEBuilder HiFi DNA Assembly Cloning Kit (New England Biolabs). Genetic constructs were transformed into the donor strain E. coli S17-1 and conjugated into B. bacteriovorus as described previously (29, 44). Double-crossover deletion mutant exconjugants were generated by sucrose suicide counter-selection and verified by Sanger sequencing. To test complementation of Δ*bd3285*, either a wild-type copy of *bd3285* or a copy of *bd3285* containing the point mutation D321A (catalytic domain mutant) was introduced into the Δ*bd3285* mutant by double crossover homologous recombination as described above and verified by Sanger sequencing. The Δ*bd3285* mutant was also complemented with *bd3285-mCherry* in *trans* on the vector pMQBAD, derived from pMQ414 (45), by cloning the *bd3285* gene plus 100 bp of flanking DNA into the vector which was then conjugated into B. bacteriovorus Δ*bd3285*.

### Construction of fluorescent fusions and overexpression strains.

To label the cytoplasm of B. bacteriovorus and thus allow visualization of predator cells within prey bdelloplasts, a fluorescent fusion of Bd0064-mCherry was introduced into B. bacteriovorus strains via single-crossover homologous recombination and maintained under kanamycin selection.

To construct a single-crossover fluorescent fusion of Bd3285-mCherry, the gene (minus the signal peptide and stop codon and with *mCherry* fused to the 3′ end) was assembled into pK18*mobsacB* using Gibson Assembly. Constructs were conjugated into B. bacteriovorus containing a double-crossover fluorescent fusion of Bd0064-mCerulean3, verified by Sanger sequencing and maintained under kanamycin selection.

To construct a strain of E. coli overexpressing Bd3285-mCherry, the *bd3285* gene (minus the stop codon and with *mCherry* fused to the 3′ end) was cloned into the vector pBAD HisA under the control of an arabinose-inducible araBAD promoter. The construct was transformed into E. coli TOP10, verified by Sanger sequencing and maintained under kanamycin selection.

### Phase contrast and epifluorescence microscopy.

Cells were pipetted onto a thin 1% Ca/HEPES agarose pad and imaged under the Plan Apo ×100 Ph3 oil objective lens (NA: 1.45) of an inverted Nikon Ti-E epifluorescence microscope. The following filters were used to acquire fluorescence images: mCherry (excitation: 555 nm, emission: 620/60 nm), DAPI: (excitation: 395 nm, emission: 435 to 485 nm), and mCerulean3 (excitation: 440 nm, emission: 470 to 490 nm). Images were captured on an Andor Neo sCMOS camera using Nikon NIS software. To overexpress Bd3285, E. coli TOP10 cells were either induced with 0.2% L-arabinose for 20 h at 37°C. Samples were then removed and placed onto a 1% agarose pad for imaging.

### Labeling of the peptidoglycan cell wall.

To visualize the prey cell wall during predation, E. coli prey were prelabeled with the fluorescent D-amino acid (FDAA) cell wall stain HADA (46) (kind gift from Dr. Erkin Kuru and Prof. Michael VanNieuwenhze, Indiana University) prior to predation. In brief, E. coli S17-1 was cultured for 16 h, adjusted to an OD_600_ = 1.0 in fresh YT medium, mixed with 500 μM HADA, and incubated at 29°C for 30 min. Cells were then microcentrifuged at 17,000 g for 5 min and the pellet washed with Ca/HEPES. The process was repeated to remove any unincorporated free HADA stain.

Predatory cultures for a semisynchronous time course were set up in a 5:4:3 ratio of 10-fold concentrated predator cells: HADA-labeled E. coli: Ca/HEPES buffer. After 30 min, 120 μL was removed and transferred to a microcentrifuge tube containing 175 μL of precooled 100% ethanol. Tube contents were mixed by inversion, incubated at −20°C for 15 min, then microcentrifuged at 17,000 g for 5 min and the pellet resuspended in 500 μL of 1× PBS. Samples were microcentrifuged again and the pellet resuspended in 5 μL of Slow-Fade (Molecular Dimensions) and stored at −20°C. For imaging, 2 μL of each sample was transferred to a microscope slide and imaged as a wet mount. HADA fluorescence was captured with the DAPI filter and a 1 s exposure time, while B. bacteriovorus Bd0064-mCherry fluorescence was captured with the mCherry filter and a 10 s exposure time.

### Time-lapse microscopy.

Time-lapse microscopy videos of B. bacteriovorus invasion into E. coli prey cells were captured under the ×100 oil objective lens (NA: 1.25) of an upright Nikon Eclipse E600 microscope. To prepare samples, 1 mL of attack-phase B. bacteriovorus and 50 μL of stationary-phase E. coli S17-1 were separately microcentrifuged at 17,000 g for 2 min and resuspended in 50 μL of Ca/HEPES. Predators and prey were mixed and transferred onto a soft 0.3% Ca/HEPES agarose pad. Time-lapse images of multiple fields of view were captured every 1 min for at least 2 h using a motorized Prior Scientific H101A XYZ stage and Hammamatsu Orca ER Camera with Simple PCR software.

### Image analysis.

The Fiji distribution of ImageJ (47) was used for image processing and analysis. Images were sharpened and smoothed and minimal adjustments were made to brightness and contrast. The MicrobeJ plug-in for Fiji (48) (v. 5.11z) was used to detect B. bacteriovorus (labeled with Bd0064-mCherry) within prey bdelloplasts and measure the different morphologies of bdelloplasts. B. bacteriovorus predators within bdelloplasts were detected in the Maxima tab using the “Bacteria” setting. Bdelloplasts were generally identified by the parameters of area: 1.0-max μm^2^, length: 0.5-max μm, width: 0.5-max μm, curvature 0 to 0.35 A.U., and circularity: 0.6 to 1.0 A.U. Circularity is defined by MicrobeJ as “4πr x area/perimeter^2^, with a value of 1.0 indicating a perfect circle.” Bdelloplasts with a circularity value of ≤0.96 A.U. were classified as nonspherical based on visual observations and the fact that approximately 0% of wild-type bdelloplasts had a circularity score of ≤0.96 A.U. The classification of nonspherical bdelloplasts allowed the proportion of spherical bdelloplasts to be quantified. All images were manually inspected to ensure that cells had been correctly detected. Bdelloplasts that either did not contain a B. bacteriovorus predator or had B. bacteriovorus cells attached to the outside (distorting shape measurements) were removed prior to analysis.

### Western blot.

Semisynchronous predation was set up for Bd3285 (WT)-mCherry and Bd3285 D312A-mCherry strains, as described above and at 30 min postmixing of predators and prey, 500 μL samples were concentrated to 100 μL by centrifugation at 17,000 × *g* for 2 min. This was followed by the addition of 40 μL of 4× loading buffer (2 mL 10% SDS, 0.5 mL 0.5% bromophenol blue, 600 μL 1M Tris pH 6.8, 350 μL water, 1.25 mL 80% glycerol, 500 μL β-mercaptoethanol) and samples were frozen at −20°C. Equivalent volumes of attack-phase B. bacteriovorus only and E. coli only controls were also collected and concentrated to 100 μL. Samples were boiled at 105°C for 5 min then 10 μL was loaded onto 4% to 20% SDS-PAGE gels with 3 μL MagicMark XP ladder (Invitrogen) or 10 μL SeeBlue Plus2 ladder (Invitrogen) for loading control gels, which were stained with QuickBlue Protein Stain (LubioScience). Gels were transferred onto a nitrocellulose membrane for 2 h at 25 V. The WesternBreeze Chemiluminescence kit (Novex) was used for immunodetection according to the manufacturer’s instructions with anti-mCherry primary antibody (Invitrogen, product no: PA5-34974, diluted 1:2,000) incubated overnight at 4°C. Images were captured via exposure to X-ray film.

### Statistical analysis.

All statistical analyses were performed in GraphPad Prism 8.0. Collected data were first tested for normality to assess whether the data exhibited a Gaussian or non-Gaussian distribution. Data sets were subsequently subjected to the most appropriate statistical test. The statistical tests applied to data sets, *P*-values, and number of biological repeats performed for each experiment are described in the figure legends.
