# Supplementary material for: An MltA-Like Lytic Transglycosylase Secreted by Bdellovibrio bacteriovorus Cleaves the Prey Septum during Predatory Invasion
Source: J Bacteriol. 2023 Apr 3;205(4):e00475-22. doi: 10.1128/jb.00475-22 (PMC10127604; doi:10.1128/jb.00475-22)
Supplement: Supplemental file 1 — Fig. S1 to S17 and Tables S1 to S3. Download jb.00475-22-s0001.docx, DOCX file, 7.0 MB [file jb.00475-22-s0001.docx]

**Supplementary information for:**

**An MltA-like lytic transglycosylase secreted by *Bdellovibrio bacteriovorus* cleaves the prey septum during predatory invasion**

Banks *et al.*


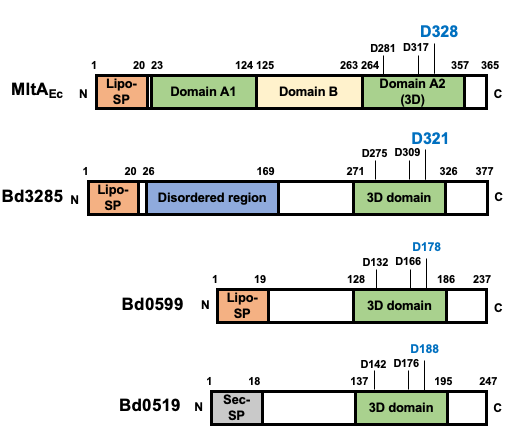


FIG S1 Domain schematics of three *B. bacteriovorus* MltA-like proteins

Schematics of predicted domains and catalytic residues for *E. coli* K12 MltA and the *B. bacteriovorus* HD100 MltA-like family proteins Bd3285, Bd0599 and Bd0519. Amino acid positions are indicated by numbers above each protein. Lipo-SP: lipoprotein signal peptide, Sec-SP: sec signal peptide. The three conserved aspartate residues within the 3D domain are labelled with the critical catalytic residue coloured in red. Schematics were drawn using information from the SignalP 5.0 prediction server (1), Pfam server (2) and IUPred3 server (3).


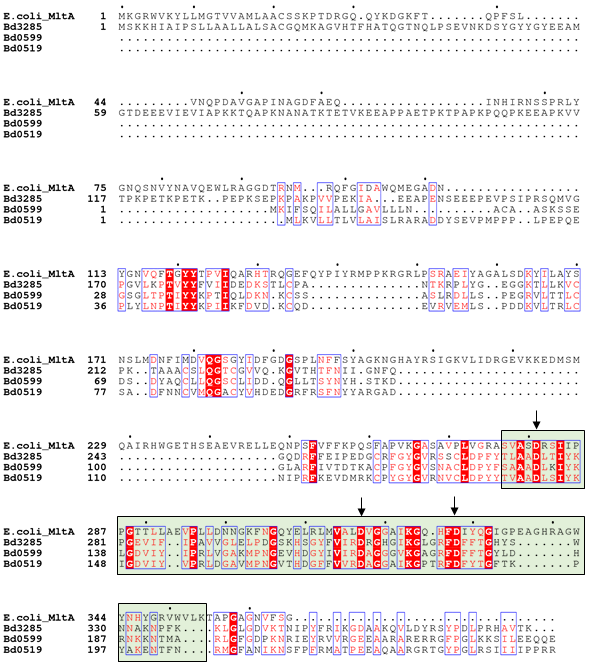


FIG S2 Sequence alignment of the *B. bacteriovorus* HD100 MltA-family proteins with *E. coli* K12 MltA

Sequence alignment of MltA from *E. coli* K12 with the MltA-like family proteins of *B. bacteriovorus* HD100: Bd3285, Bd0599 and Bd0519. Green boxed region: the predicted catalytic 3D domain of MltA from *E. coli*. Black arrows: the three conserved aspartate residues of the 3D domain. Red character: similarity across some proteins. Blue frame: similarity across all proteins. White character within a red box: strict identity conserved across all proteins. The sequence alignment was generated with Clustal Omega (4) and visualized with ESPript 3.0 (5).


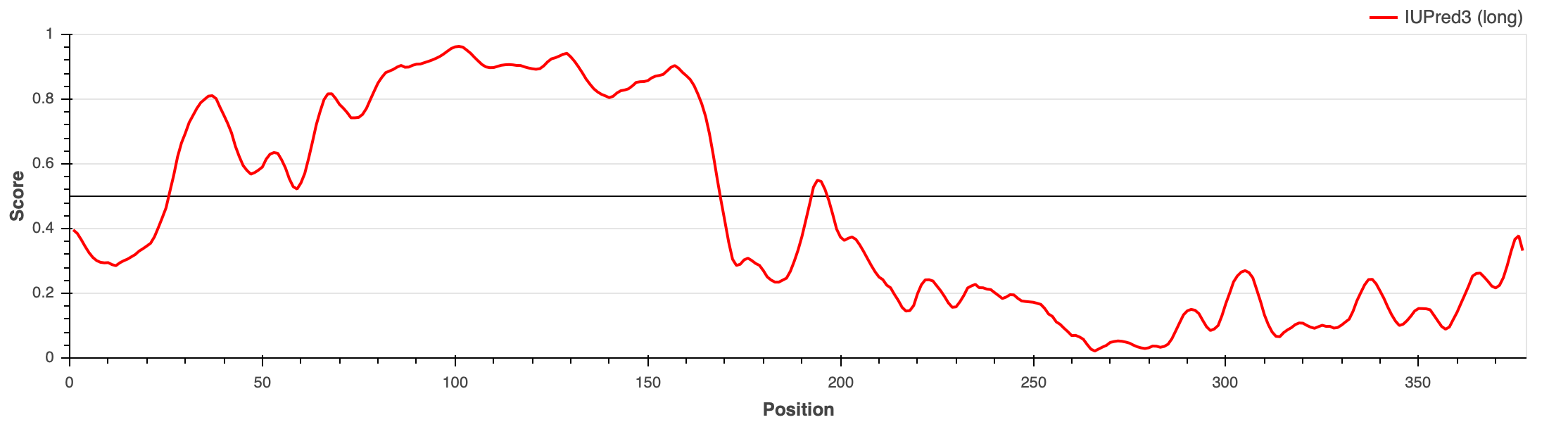


FIG S3 Bd3285 is predicted to contain a long, disordered N-terminus

Prediction of disordered regions within the Bd3285 protein obtained using IUPRed3 (3). Disorder is indicated where the red line is above the baseline (amino acids 26-169).


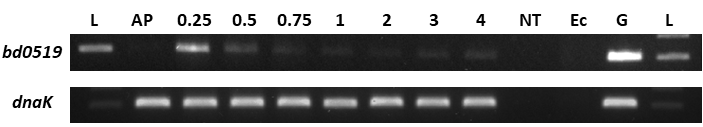


FIG S4 *B. bacteriovorus* *bd0519* is upregulated at 0.25-0.5 h during predatory invasion

Reverse-transcriptase PCR (RT-PCR) performed on RNA isolated from different timepoints across the life cycle of *B. bacteriovorus* HD100. Primers were designed to amplify an approximately 100 bp product internal to *bd0519* or *dnaK* (known constitutively transcribed control gene). L: 100 bp DNA ladder, AP: attack-phase, 0.25-4: h since predators and prey were mixed, NT: no-template control, Ec: *E. coli* S17-1 RNA, G: *B. bacteriovorus* HD100 genomic DNA. Image is representative of at least two biological repeats for each gene.

**
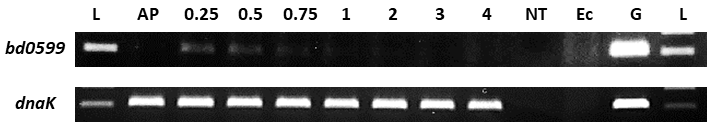
**

FIG S5 *B. bacteriovorus* *bd0599* is upregulated at 0.25-0.5 h during predatory invasion

Reverse-transcriptase PCR (RT-PCR) performed on RNA isolated from different timepoints across the life cycle of *B. bacteriovorus* HD100. Primers were designed to amplify an approximately 100 bp product internal to *bd0599* or *dnaK* (known constitutively transcribed control gene). L: 100 bp DNA ladder, AP: attack-phase, 0.25-4: h since predators and prey were mixed, NT: no-template control, Ec: *E. coli* S17-1 RNA, G: *B. bacteriovorus* HD100 genomic DNA. Image is representative of at least two biological repeats for each gene.


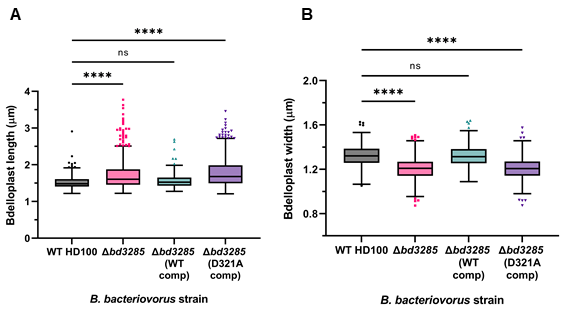


FIG S6 Length and width of bdelloplasts invaded by different *B. bacteriovorus* strains

Length **(A)** and width **(B)** of *E. coli* S17-1 prey bdelloplasts invaded by *B. bacteriovorus* wild-type (WT HD100), Δ*bd3285*, Δ*bd3285* (WT comp) and Δ*bd3285* (D321A comp) strains. Box: 25^th^-75^th^ percentiles, line: median, whiskers: Tukey. n = 234-670 total cells analyzed at the 1 h predatory timepoint from three biological repeats. ns: non-significant; ****: p<0.0001 (**A**: Kruskal-Wallis test with Dunn’s multiple comparisons tests; **B**: One-way ANOVA with Tukey’s multiple comparisons tests).


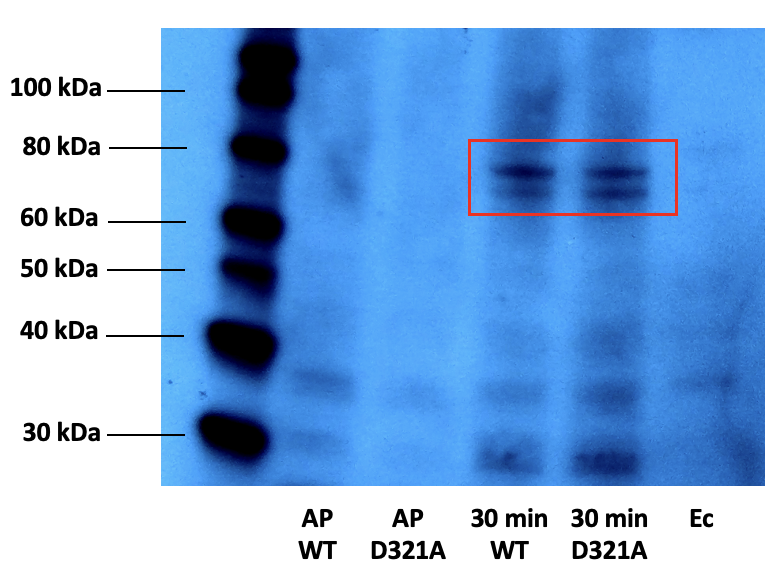


FIG S7 Western blot to confirm stability of the Bd3285 (D321A) protein

Western blot, using anti-mCherry antiserum, of Bd3285 (WT)-mCherry and Bd3285 (D321A)-mCherry single-crossover fluorescent fusions expressed in *B. bacteriovorus* HD100 during predation. Ladder: Magicmark XP (Thermofisher), AP: attack-phase *B. bacteriovorus* strains, 30 min: 30 min post-mixing of predators and prey, WT: Bd3285 (WT)-mCherry, D321A: Bd3285 (D321A)-mCherry, Ec: *E. coli* only control. Predators and prey were mixed, in identical ways, for a semi-synchronous infection (see methods) and samples were collected after 30 min. Samples were probed with polyclonal anti-mCherry antiserum (Invitrogen) Bands are visible at the predicted size of Bd3285-mCherry (66 kDa) but also at ~75 kDa (red box), suggesting a possible post-translational modification. The image is representative of three independent repeats and similar amounts of wild-type and D321A protein were observed each time, suggesting that the D321A version is stable.


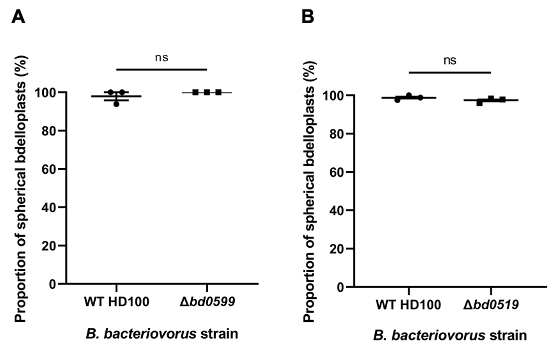


FIG S8 Neither Bd0599 nor Bd0519 have roles in bdelloplast shape transformation

Proportion of spherical bdelloplasts observed at 1 h during predation on *E. coli* S17-1 by either Δ*bd0599* **(A)** or Δ*bd0519* **(B)** compared to wild-type *B. bacteriovorus*. Bdelloplasts were classified as spherical if they had a circularity value of >0.96 A.U. Error bars present SE of the mean. ns: non-significant. For A,

n=197-199 total cells analyzed from three biological repeats and p=0.37 (two-tailed unpaired t-test). For B, n=255-269 cells analyzed from three biological repeats and p=0.24 (two-tailed unpaired t-test).


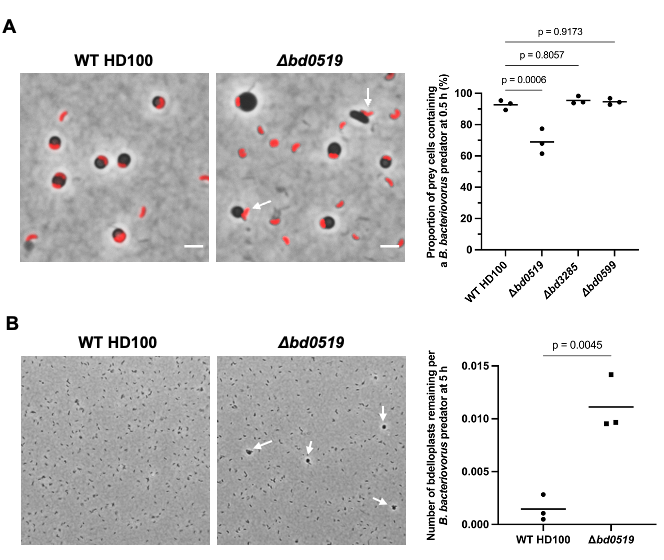


FIG S9 A *bd0519* deletion mutant is delayed in prey entry and thus prey exit

**(A)** Left: representative images of predation on *E. coli* S17-1 prey by wild-type or Δ*bd0519* *B. bacteriovorus* predator cells at the timepoint of 0.5 h. Arrows indicate prey cells that have not yet been invaded by Δ*bd0519*. The predator cytoplasm is labelled by the cytoplasmic fusion of Bd0064-mCherry to visualize predator cells within prey. Right: proportion of prey cells containing a *B. bacteriovorus* predator at 0.5 h for wild-type, Δ*bd0519*, Δ*bd3285* and Δ*bd0599* strains. Data points are from 3 biological repeats and were analyzed by a one-way ANOVA with Tukey’s multiple comparison test. Line indicates the mean. **(B)** Left: representative images of predation on *E. coli* S17-1 prey by wild-type or Δ*bd0519* *B. bacteriovorus* predator cells at the timepoint of 5 h (when all bdelloplasts should have lysed). Arrows indicate prey cells that have not yet been lysed by Δ*bd0519*. Right: bdelloplasts remaining at 5 h per *B. bacteriovorus* free-swimming predator cell (p=0.0045; two-tailed unpaired t-test). Data are from 3 biological repeats. Line indicates the mean.

**
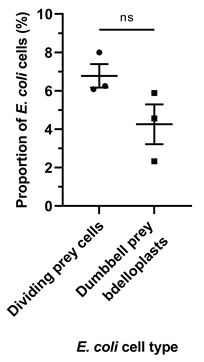
**

FIG S10 Proportion of dividing *E. coli* cells and the proportion of dumbbell bdelloplasts

Proportion of *E. coli* cells in the process of cell division prior to predation by *B. bacteriovorus* Δ*bd3285* compared to the proportion of *E. coli* dumbbell-shaped prey bdelloplasts observed 1 h into predation with *B. bacteriovorus* Δ*bd3285*. Dividing cells and dumbbell-shaped bdelloplasts were manually identified by the presence of a constriction in width at the mid-cell. Error bars represent the SE of the mean. ns: p=0.10 (two-tailed Mann-Whitney test). Data are from three biological repeats.


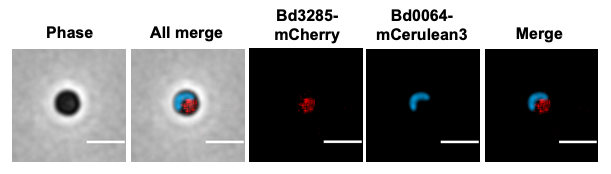


FIG S11 Bd3285-mCherry does not co-localize with *B. bacteriovorus* predators inside prey bdelloplasts

Fluorescence microscopy images of *B. bacteriovorus* HD100 containing both a single-crossover fusion of Bd3285-mCherry and a double-crossover fusion of Bd0064-mCerulean3 during predation on *E. coli* S17-1. Scale bars = 2 μm. Images were acquired 1.5 h after predator-prey mixing and are representative of three biological repeats.


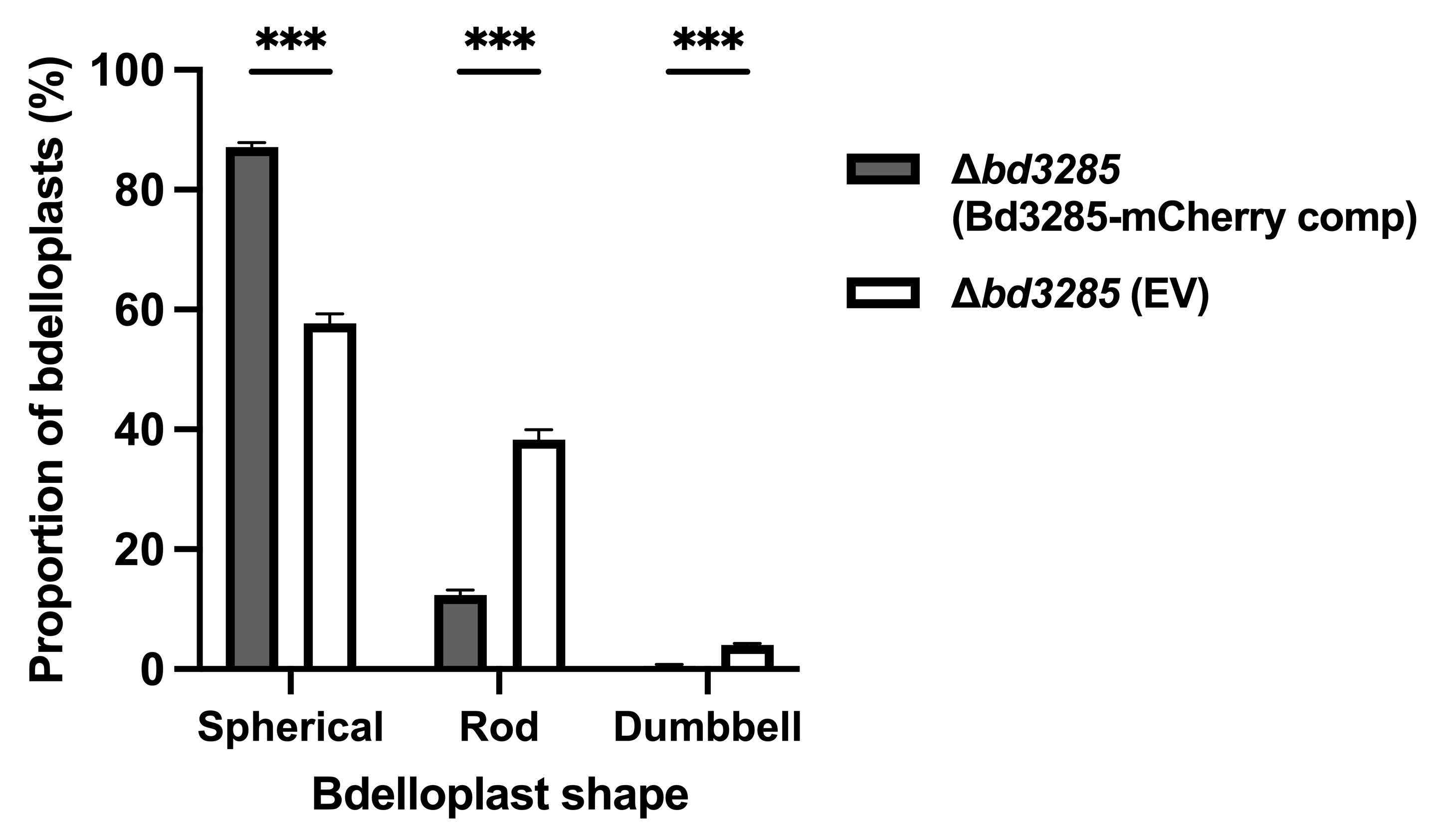


FIG S12 Bd3285-mCherry, expressed *in trans,* can complement the phenotype of the Δ*bd3285* mutant

Complementation of the Δ*bd3285* mutant strain by supplying Bd3285-mCherry *in trans* from the plasmid pMQBAD restored rounding of prey bdelloplasts during predation, versus a PMQBAD empty vector control (EV), confirming that the Bd3285-mCherry fluorescent fusion protein is still functional. Error bars present SE of the mean. n = 467-581 total cells analysed from three biological repeats. ***: p<0.001 (unpaired t-tests).


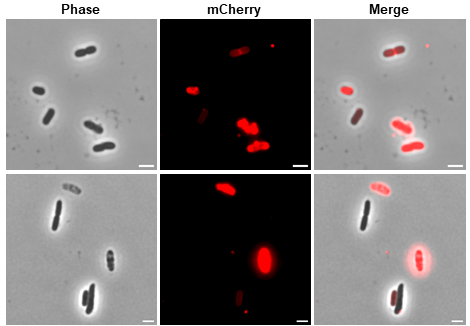


FIG S13 Examples of heterogeneity in Bd3285-mCherry expression from *E. coli* TOP10

Fluorescence microscopy images of stationary-phase *E. coli* TOP10 induced with 0.2% arabinose to overexpress Bd3285-mCherry. Scale bars = 2 μm. Images are representative of three biological repeats.


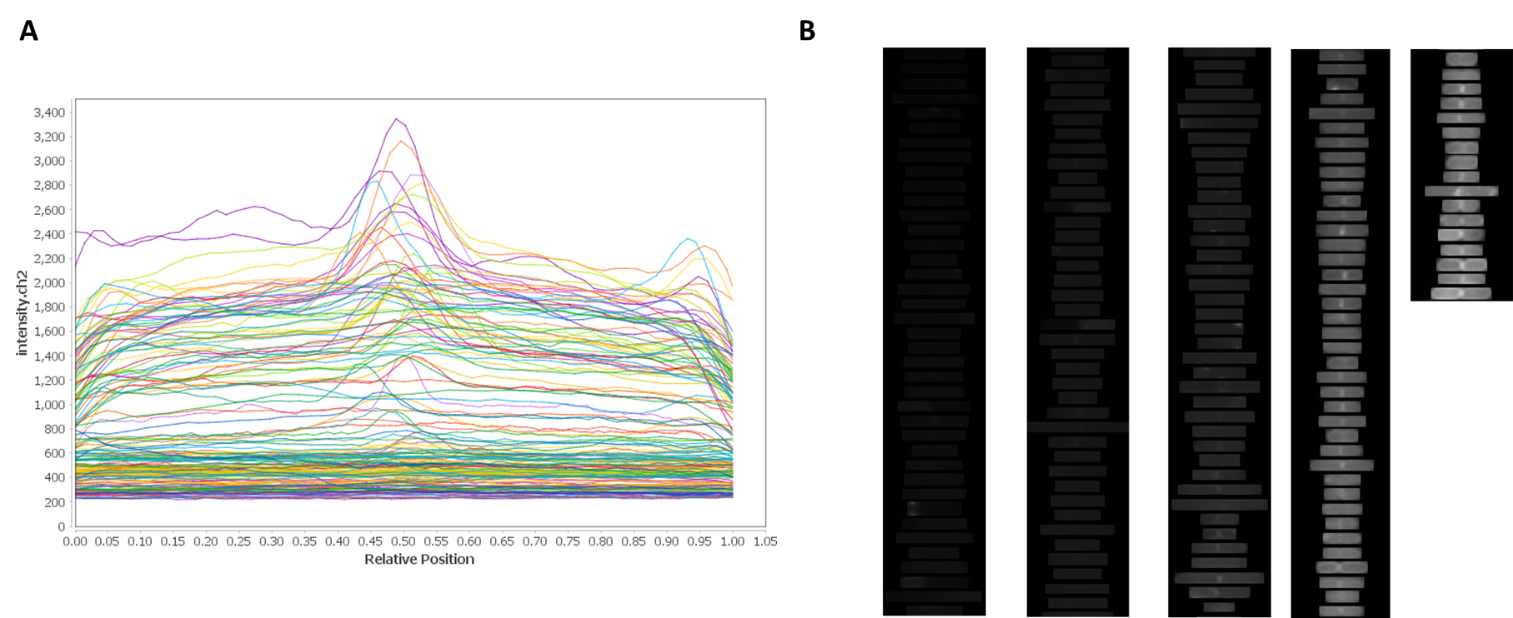


FIG S14 Bd3285-mCherry fluorescence intensity during heterologous expression in *E. coli* cells

**(A)** Fluorescence intensity of Bd3285-mCherry signal measured across the length of *E. coli* cells using MicrobeJ software (6). Cells with a detectible constriction (measured with default settings in the features option in MicrobeJ) were chosen and manually inspected. Cells were rejected for analysis if the average fluorescence intensity was over 3500 AU or if the cells appeared damaged. All cell lengths were normalized. A peak in fluorescence intensity at the midcell can be observed. The extreme variability in total signal meant that statistical analyses could not be applied to these data. **(B)** Profiles of Bd3285-mCherry fluorescent signal across all of the cells analyzed shows a concentration of signal intensity in the midcell. n=172 cells from three biological repeats.


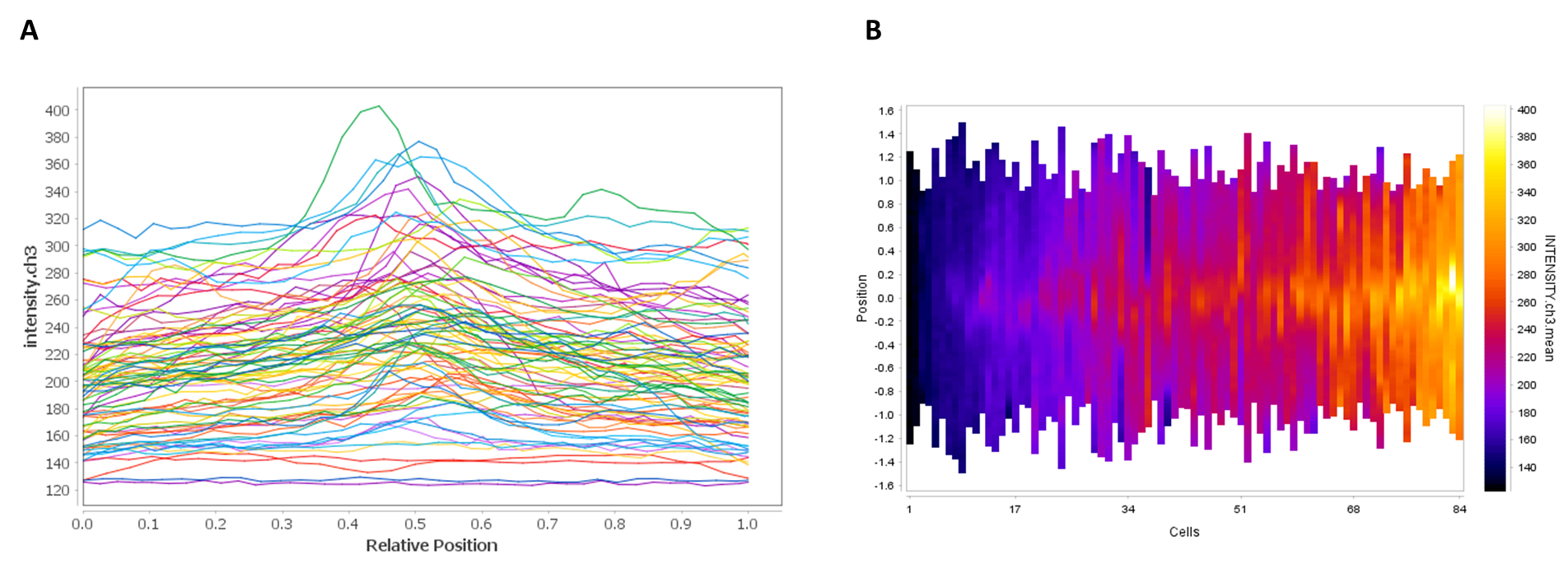


FIG S15 HADA fluorescence intensity plots of prey cells with a detectable midcell constriction, invaded by *B. bacteriovorus* Δ*bd3285.*

**(A)** Fluorescence intensity of HADA signal measured across the length of *E. coli* prey dumbbell bdelloplasts invaded by the *B. bacteriovorus* Δ*bd3285* mutant. Dumbbell bdelloplasts with a midcell constriction (detected by the features option in MicrobeJ) were identified and manually inspected. **(B)** Demograph of HADA fluorescences across all individual cells (n=94), ordered by total mean fluorescence, showing a midcell intensity peak in the majority of cells.


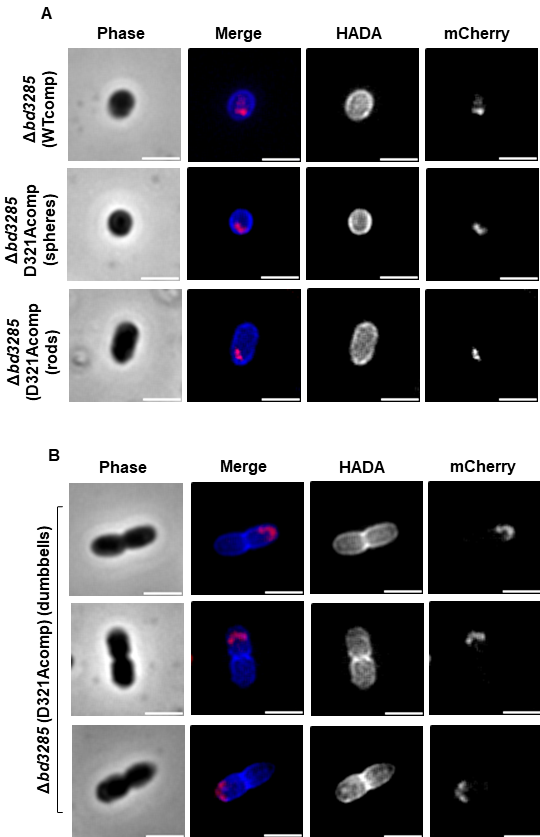


FIG S16 Complementation of Δ*bd3285* with a WT copy of *bd3285* restores prey septum cleavage but a D321A catalytic point mutant copy does not

Fluorescence microscopy images of *E. coli* S17-1 prey invaded by either *B. bacteriovorus* Δ*bd3285* (WT comp) or Δ*bd3285* (D321A comp). The *E. coli* PG wall was pre-labelled with the blue D-amino acid HADA prior to predation. *B. bacteriovorus* predator strains contain a Bd0064-mCherry fusion to label the predator cytoplasm and allow visualisation of predators inside prey. Samples were fixed for imaging 30 min after predator-prey mixing. **(A)** Spherical bdelloplast invaded by a Δ*bd3285* (WT comp) predator (top row), and spherical and rod-shaped bdelloplasts invaded by Δ*bd3285* (D321A comp) (middle and bottom rows, respectively). **(B)** Examples of dumbbell-shaped bdelloplasts invaded by Δ*bd3285* (D321A comp) which still contain a septum. Scale bars = 2 µm and images are representative of three biological repeats.

**
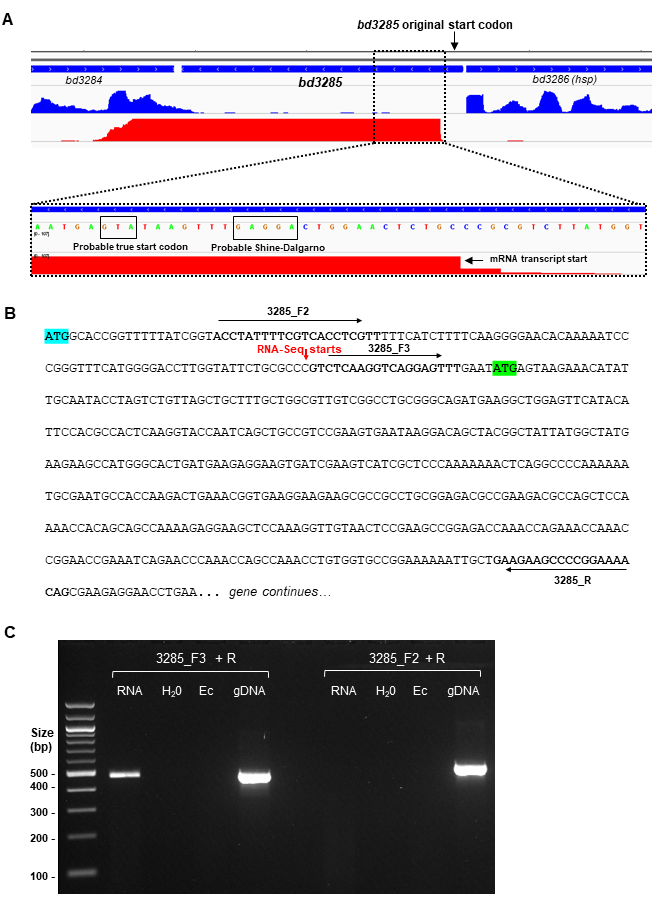
**

FIG S17 Re-annotation of the *bd3285* start codon

**(A)** *B. bacteriovorus* HD100 RNA-Seq reads from a 15 min predatory cycle timepoint aligned to the genome of *B. bacteriovorus* HD100 with Rockhopper. Reads (red block) show the transcriptional initiation site of *bd3285* (blue) which is encoded on the reverse strand. Transcription begins 106 bp into the gene sequence, indicating that the start codon was mis-annotated. The probable true Shine-Dalgarno site (AGGAG) and start codon (ATG) are annotated. The DNA sequence was reverse-complemented for ease of viewing. Data were visualized in Integrative Genomics Viewer. **(B)** 5’ end of the *bd3285* gene. The originally annotated ATG start codon is highlighted in blue, the more probable true ATG start codon is highlighted in green and the RNA-Seq transcription start site is indicated by a red arrow. **(C)** RT-PCR to test if the start codon of *bd3285* was mis-annotated using the primers 3285_F3 with 3285_R or 3285_F2 with 3285_R. The annealing site of each primer is indicated in a. No product was detected using the 3285_F2 primer but a 481 bp product was detected using 3285_F3, indicating that the RNA-Seq data is correct and that the original ATG start codon was mis-annotated. RNA: *B. bacteriovorus* HD100 15 min RNA, H_2_O: negative water control, Ec: *E. coli* S17-1 negative control, gDNA: *B. bacteriovorus* HD100 genomic DNA positive control. The RT-PCR is representative of two biological repeats.

**Table S1 Primers used in this research study**

| **Primer** | **Sequence (5’ 🡪 3’)** | **Purpose** |
| --- | --- | --- |
| 3285_F1 | CGGAGACCAAACCAGAAACC | Transcriptional pattern of *bd3285* across the predatory cycle |
| 3285_R | CTGTTTTCCGGGGCTTCTTC |  |
| 3285_F2 | ACCTATTTTCGTCACCTCGTT | Testing the transcriptional start site of *bd3285* |
| 3285_F3 | GTCTCAAGGTCAGGAGTTT |  |
| 3285_R | CTGTTTTCCGGGGCTTCTTC |  |
| 0599_F | GCCCAATGGTGAGGTTCATG | Transcriptional pattern of *bd0599* across the predatory cycle |
| 0599_R | TTGCGATGATCCAGGTAGCC |  |
| 0519_F | GGTCCGGGTTGAAATGCTTT | Transcriptional pattern of *bd0519* across the predatory cycle |
| 0519_R | CGTGAACATAACAGGCCCCT |  |
| dnaK_F | TGAGGACGAGATCAAACGTG | Transcriptional pattern of *dnaK* across the predatory cycle |
| dnaK_R | AAACCAGGTTGTCGAGGTTG |  |
| 3285_up_F | CGTTGTAAAACGACGGCCAGTGCCAAATTCA  AAATCCGTCTCATTGC | Amplifies 1 kb of DNA upstream of *bd3285* for making KO |
| 3285_up_R | GGGACTATTTGGTACTCATATTCAAACTCCT  GACC |  |
| 3285_down_F | TTGAATATGAGTACCAAATAGTCCCGCGTG | Amplifies 1 kb of DNA downstream of *bd3285* for making KO |
| 3285_down_R | GGAAACAGCTATGACCATGATTACGTGCGG  CAAACTCAATGAG |  |
| 0599_up_F | CGTTGTAAAACGACGGCCAGTGCCATCAAA  ATTGCCCGATCTCAAG | Amplifies 500 bp of DNA upstream of *bd0599* for making KO |
| 0599_up_R | CTGGGAAACCTCTTTCATGTTTGGGCCCTC |  |
| 0599_down_F | CCCAAACATGAAAGAGGTTTCCCAGGACTTA  AAAAG | Amplifies 500 bp of DNA downstream of *bd0599* for making KO |
| 0599_down_R | GGAAACAGCTATGACCATGATTACGATCACA  TGCGGAAAAAGTTTTG |  |
| 0519_up_F | cgttgtaaaacgacggccagtgccaGGAATCTCATCAGCACGC | Amplifies 1 kb of DNA upstream of *bd0519* for making KO |
| 0519_up_R | ccatgcggaaaggCAACATGGCCCCATACTAAC |  |
| 0519_down_F | tggggccatgttgCCTTTCCGCATGGCGACG | Amplifies 1 kb of DNA downstream of *bd0519* for making KO |
| 0519_down_R | ggaaacagctatgaccatgattacgCCAACGGACGTGAATGCAAAAAG |  |
| 3285_up_F | CGTTGTAAAACGACGGCCAGTGCCAAATTCA  AAATCCGTCTCATTGC | Complementation of Δ*bd3285* with wild-type *bd3285* gene |
| 3285_down_R | GGAAACAGCTATGACCATGATTACGTGCGG  CAAACTCAATGAG |  |
| 3285_up_F | CGTTGTAAAACGACGGCCAGTGCCAAATTCA  AAATCCGTCTCATTGC | Complementation of Δ*bd3285* with D321A point-mutated *bd3285* gene |
| 3285_D321A_R | GTAAAGAACGCAAATCGGCCC |  |
| 3285_D321A_F | GGGCCGATTTGCGTTCTTTAC |  |
| 3285_down_R | GGAAACAGCTATGACCATGATTACGTGCGG  CAAACTCAATGAG |  |
| 3285genmCh_F | CGTTGTAAAACGACGGCCAGTGCCATGCGG  GCAGATGAAGGCTG | Construction of Bd3285-mCherry single-crossover fusion |
| 3285genmCh_R | CTTGCTCACCATTTTGGTGACCGCGTGACG |  |
| 3285_mCh_F | CGCGGTCACCAAAATGGTGAGCAAGGGCGA  G |  |
| 3285_mCh_R | GGAAACAGCTATGACCATGATTACGTTACTT  GTACAGCTCGTCCATG |  |
| 3285_pBAD_F | TTTTGGGCTAACAGGAGGAATTAACCATGAG  TAAGAAACATATTGCAATAC | Construction of Bd3285-mCherry fusion in pBAD vector |
| 3285mChpBADR | CTTGCTCACCATTTTGGTGACCGCGTGACG |  |
| 3285mChpBADF | CGCGGTCACCAAAATGGTGAGCAAGGGCGA  G |  |
| 3285_pBAD_R | ACCCATTTGCTGTCCACCAGTCATGTTACTT  GTACAGCTCGTCCATG |  |

**Table S2 Plasmids used in this research study**

| **Plasmid** | **Description** | **Source** |
| --- | --- | --- |
| pK18*mobsacB* | Suicide vector (kanR, *lacZα*, *sacB*) used for crossovers into the *B. bacteriovorus* genome | (7) |
| pAKF56 | Template for *mCherry* gene | (8) |
| pmCerulean3-N1 | Template for *mCerulean3* gene | Addgene (54730) |
| pBAD HisA | pBAD HisA vector, *ara*BAD promoter, *ara*C, kanamycin resistance | (9) |
| pMQBAD | Complementation vector for *B. bacteriovorus* derived from pMQ414 | This lab. Derived from (10) |
| pK18_3285KO | Upstream and downstream fragments of *bd3285* to make KO | This study |
| pK18_0599KO | Upstream and downstream fragments of *bd0599* to make KO | This study |
| pK18_0519KO | Upstream and downstream fragments of *bd0519* to make KO | This study |
| pK18_3285_WTcomp | Upstream and downstream fragments of *bd3285* and wild-type *bd3285* gene for complementation | This study |
| pK18_3285_D321Acomp | Upstream and downstream fragments of *bd3285* and D321A point-mutated *bd3285* gene for complementation | This study |
| pK18_3285-mChSXO | *bd3285* gene fused to *mCherry* used to generate a single-crossover fusion in *B. bacteriovorus* | This study |
| pBAD3285-mCh | pBAD plasmid containing wild-type *bd3285* gene fused to *mCherry* under an arabinose-inducible promoter | This study |

**Table S3 Strains used in this research study**

| **Strain** | **Description** | **Source** |
| --- | --- | --- |
| *E. coli* DH5α | *E. coli* cloning strain (F- endA1 hsdR17 (rk -mk -) supE44 thi-1 recA1 gyrA (NaIr) relA1 D(lacIZYA-argF) U169 deoR (80dlacD (lacZ)M15)) | (11) |
| *E. coli* S17-1 | *E. coli* strain (thi, pro, hsdR-, hsdM+, recA; integrated plasmid RP4- Tc::Mu-Kn::tn) | (12) |
| *E. coli* S17-1: pZMR100 | *E. coli* strain containing the plasmid pZMR100 (kanR) | (13) |
| *E. coli* TOP10 | *E. coli* strain for arabinose-inducible gene expression (F- mcrA Δ(mrr-hsdRMSmcrBC) φ80lacZΔM15 ΔlacX74 deoR recA1 araD139 Δ(araA-leu)7697 galU galK rpsL endA1 nupG) | Gift from Dr Ruth Griffin |
| *B. bacteriovorus* HD100 | *B. bacteriovorus* Type strain, genome-sequenced, wild-type | (14) |
| HD100 + Bd0064-mCherry SXO | *B. bacteriovorus* HD100 containing a single-crossover fusion of Bd0064-mCherry | This study |
| HD100 Δ*bd3285* | *B. bacteriovorus* HD100 containing an in-frame silent deletion of *bd3285* | This study |
| HD100 Δ*bd3285* + Bd0064-mCherry SXO | *B. bacteriovorus* HD100 containing an in-frame silent deletion of *bd3285* and a single-crossover fusion of Bd0064-mCherry | This study |
| HD100 Δ*bd0599* | *B. bacteriovorus* HD100 containing an in-frame silent deletion of *bd0599* | This study |
| HD100 Δ*bd0519* | *B. bacteriovorus* HD100 containing an in-frame silent deletion of *bd0519* | This study |
| HD100 Δ*bd3285* WTcomp | *B. bacteriovorus* HD100 containing a double-crossover homologous re-integration of the wild-type *bd3285* gene | This study |
| HD100 Δ*bd3285* WTcomp + Bd0064-mCherry SXO | *B. bacteriovorus* HD100 containing a double-crossover homologous re-integration of the wild-type *bd3285* gene and a single-crossover fusion of Bd0064-mCherry | This study |
| HD100 Δ*bd3285* D321Acomp | *B. bacteriovorus* HD100 containing a double-crossover homologous re-integration of a D321A point-mutated *bd3285* gene | This study |
| HD100 Δ*bd3285* D321Acomp + Bd0064-mCherry SXO | *B. bacteriovorus* HD100 containing a double-crossover homologous re-integration of a D321A point-mutated *bd3285* gene and a single-crossover fusion of Bd0064-mCherry | This study |
| HD100 Bd3285-mCherry SXO + Bd0064-mCerulean3 DXO | *B. bacteriovorus* HD100 containing a single-crossover fusion of Bd3285-mCherry and a double-crossover fusion of Bd0064-mCerulean3 | This study |
| *E. coli* TOP10 Bd3285-mCherry | *E. coli* TOP10 expressing Bd3285-mCherry on the pBAD plasmid | This study |

**References**

1. Almagro Armenteros JJ, Tsirigos KD, Sonderby CK, Petersen TN, Winther O, Brunak S, von Heijne G, Nielsen H. 2019. SignalP 5.0 improves signal peptide predictions using deep neural networks. Nat Biotechnol 37:420-423.

2. Sonnhammer EL, Eddy SR, Durbin R. 1997. Pfam: a comprehensive database of protein domain families based on seed alignments. Proteins 28:405-20.

3. Erdos G, Pajkos M, Dosztanyi Z. 2021. IUPred3: prediction of protein disorder enhanced with unambiguous experimental annotation and visualization of evolutionary conservation. Nucleic Acids Res 49:W297-W303.

4. Madeira F, Park YM, Lee J, Buso N, Gur T, Madhusoodanan N, Basutkar P, Tivey ARN, Potter SC, Finn RD, Lopez R. 2019. The EMBL-EBI search and sequence analysis tools APIs in 2019. Nucleic Acids Res 47:W636-W641.

5. Robert X, Gouet P. 2014. Deciphering key features in protein structures with the new ENDscript server. Nucleic Acids Res 42:W320-4.

6. Ducret A, Quardokus EM, Brun YV. 2016. MicrobeJ, a tool for high throughput bacterial cell detection and quantitative analysis. Nat Microbiol 1:16077.

7. Schafer A, Tauch A, Jager W, Kalinowski J, Thierbach G, Puhler A. 1994. Small mobilizable multi-purpose cloning vectors derived from the *Escherichia coli* plasmids pK18 and pK19: selection of defined deletions in the chromosome of *Corynebacterium glutamicum*. Gene 145:69-73.

8. Fenton AK, Kanna M, Woods RD, Aizawa SI, Sockett RE. 2010. Shadowing the actions of a predator: backlit fluorescent microscopy reveals synchronous nonbinary septation of predatory *Bdellovibrio* inside prey and exit through discrete bdelloplast pores. J Bacteriol 192:6329-35.

9. Guzman LM, Belin D, Carson MJ, Beckwith J. 1995. Tight regulation, modulation, and high-level expression by vectors containing the arabinose PBAD promoter. J Bacteriol 177:4121-30.

10. Mukherjee S, Brothers KM, Shanks RMQ, Kadouri DE. 2015. Visualizing *Bdellovibrio bacteriovorus* by using the tdTomato fluorescent protein. Appl Environ Microbiol 82:1653-1661.

11. Simon R, Preifer U, Puhler A. 1983. A broad host range mobilisation system for *in vivo* genetic engineering: transposon mutagenesis in gram negative bacteria. Biotechnology 9:184-191.

12. Hanahan D. 1983. Studies on transformation of *Escherichia coli* with plasmids. J Mol Biol 166:557-80.

13. Rogers M, Ekaterinaki N, Nimmo E, Sherratt D. 1986. Analysis of Tn7 transposition. Mol Gen Genet 205:550-6.

14. Rendulic S, Jagtap P, Rosinus A, Eppinger M, Baar C, Lanz C, Keller H, Lambert C, Evans KJ, Goesmann A, Meyer F, Sockett RE, Schuster SC. 2004. A predator unmasked: life cycle of *Bdellovibrio bacteriovorus* from a genomic perspective. Science 303:689-92.
